# Patent Foramen Ovale: Epidemiology, Risk Factors, Pathophysiology, Clinical Features, Diagnosis, and Management

**DOI:** 10.1002/mco2.70957

**Published:** 2026-09-06

**Authors:** Linlin Meng, Xiaotong Zhang, Guangrui Sun, Honggang Dai, Xiao Meng, Cheng Zhang, Yun Zhang

**Affiliations:** ^1^ State Key Laboratory for Innovation and Transformation of Luobing Theory; Key Laboratory of Cardiovascular Remodeling and Function Research of MOE, NHC, CAMS and Shandong Province; Department of Cardiology, Qilu Hospital of Shandong University; Cheeloo College of Medicine Shandong University Jinan Shandong China

**Keywords:** cryptogenic stroke, patent foramen ovale, PFO management, transcatheter PFO closure

## Abstract

Patent foramen ovale (PFO) is a highly prevalent cardiac abnormality, affecting approximately 25% of the population worldwide. Although a PFO may be discovered incidentally and remain asymptomatic throughout life, it can also be a conduit for paradoxical emboli or vasoactive substances and has been associated with neurological disorders, including cryptogenic stroke, transient ischemic attack, migraine, epilepsy, as well as systemic embolism, hypoxemic disorders, and decompression sickness, particularly in individuals with high‐risk anatomy or traditional cardiovascular risk factors. However, accurate identification of pathogenic PFO remains a challenge in current clinical practice, and the indications, timing, and long‐term safety of PFO closure remain controversial. In this review, we summarize the epidemiology, risk factors, pathophysiological mechanisms, clinical manifestations, diagnostic strategies, and management of PFO, with particular emphasis on risk stratification, emerging imaging tools, novel closure devices, procedure‐related complications, and the application of closure in special populations. In addition, we focused on the current evidence regarding when and who may benefit from PFO intervention and the surveillance of postprocedural complications. Overall, this review provides a comprehensive overview of the current findings and future perspectives for the management of PFO.

## Graphical abstract

The clinical manifestations of PFO. Although the majority of PFOs are benign and asymptomatic, they can present with a variety of clinical manifestations, or coexisting with other diseases and aggravating their symptoms, including cerebral diseases (such as CS, epilepsy, TIA, or migraine), systemic embolism (such as embolism in renal artery, coronary artery, retinal artery, visceral, or peripheral artery), hypoxemic diseases (such as COPD, OSA, or pulmonary embolism), and DCS in divers. Abbreviations: CS, cryptogenic stroke; TIA, transient ischemic attack; COPD, chronic obstructive pulmonary disease; OSA, obstructive sleep apnea; DCS, decompression sickness.

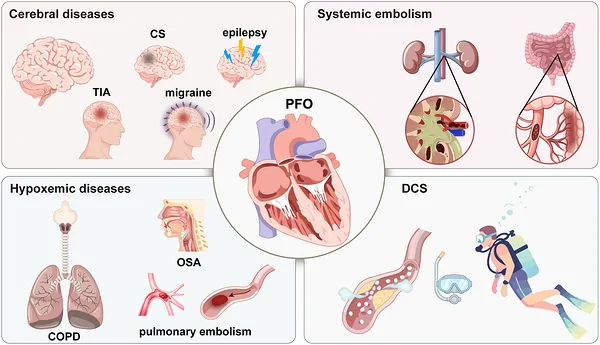

## 1 Introduction

Patent foramen ovale (PFO) is one of the most common congenital cardiac abnormalities with fetal origin, derived from the failure of fusion between the septum primum and septum secundum after birth. Although it is present in approximately 25% of the general population and remains asymptomatic throughout life, emerging evidence has demonstrated that a PFO is not merely an incidental anatomical defect [1]. As early as 1877, Cohnheim first described a case of a fatal cerebral embolism in a young woman, proposing that it resulted from a venous thrombus paradoxically entering the arterial circulation through a large PFO [2]. Despite this observation, it was not until 1988 that Lechat et al. first demonstrated the association between PFO and cryptogenic stroke (CS), especially among young patients [3]. Since then, with the advances in cardiovascular imaging and the recognition of paradoxical embolism, PFO has gradually been considered as an important contributor to various neurological and systemic disorders, transforming PFO from an incidental anatomical finding into a clinically relevant factor [4].

Over the past three decades, there has been great progress in the epidemiology, anatomical characteristics, and pathophysiological mechanisms of PFOs. The causal relationship between PFO‐mediated paradoxical emboli and ischemic stroke has been established, with emerging evidence suggesting that high‐risk anatomical features, including large right‐to‐left shunts (RLS), atrial septal aneurysm (ASA), long tunnel morphology, and embryonic remnant structures, further amplify the PFO‐related embolic risk and recurrent cardiovascular events [5, 6, 7, 8]. Transcatheter closure of PFO in patients with presumed paradoxical emboli was first reported in 1992, exhibiting little morbidity and reducing the risk of recurrence [9]. Since then, based on the evidence from several large‐scale and multicenter randomized controlled trials (RCTs) [10, 11, 12], the transcatheter PFO closure has evolved into a secondary prevention strategy for patients with a PFO‐related stroke, representing a major milestone in the field of PFO [13]. Beyond stroke, PFO has also been implicated in migraine, systemic embolism, epilepsy, hypoxemic syndromes, and other clinical conditions, although its causal role remains incompletely understood.

More recently, studies on PFO have entered a new stage extending beyond demonstrating the association between PFO and stroke. During the past 5 years, substantial progress has been made in new technologies assisting PFO detection, advanced imaging equipment, and multimodel risk stratification strategies. Meanwhile, novel transcatheter devices such as biodegradable occluders and suture systems have been applied in clinical practice [14, 15]. In addition, long‐term postclosure management, including monitoring adverse events such as arrhythmias and residual shunt, and optimizing postoperative antithrombotic strategies, has shifted PFO management toward precision medicine [16]. Despite these advances, several important clinical challenges remain unresolved. One major challenge in clinical practice is to identify the pathogenic PFO from incidental findings, particularly in the context that PFO is highly prevalent in the general population and the majority of associated diseases are driven by multiple factors. In addition, controversies persist regarding PFO closure in candidate selection, timing of intervention, management in special populations, long‐term antithrombotic strategies, and surveillance for postprocedural complications. Importantly, older adults (>60 years old) and pediatric populations (<18 years old) have typically been excluded from prior RCTs, leaving a conclusive determination about the role of PFO in these populations still lacking.

Given the rapidly evolving evidence and developments regarding PFO, an update and comprehensive review is warranted. In this study, we summarize the current understanding regarding the epidemiology, risk factors, pathophysiology, clinical features, diagnosis, and management of PFO. In particular, we highlight recent findings in the past 2 years on evolving diagnostic approaches, the indication for transcatheter closure, novel devices, procedure‐related complications, and long‐term management strategies. Particular attention is given to special populations such as elderly individuals, pediatric patients, pregnant women, and athletes, and future directions toward individualized and precision‐based management.

## 2 Epidemiology

During embryonic development, the foramen ovale is a crucial passage in the interatrial septum, allowing oxygen‐rich blood from the placenta to bypass the fetal lungs and enter systemic circulation. This essential fetal structure plays an indispensable role in normal growth and development. In most individuals, the foramen ovale spontaneously closes within the first year after birth, driven by the development of pulmonary circulation and increased left atrial pressure. However, in some cases, the foramen ovale fails to close and remains patent into adulthood, a condition known as PFO. It is estimated that approximately one‐quarter of adults have a PFO, which remains asymptomatic in the vast majority of individuals. As a result, PFO is typically discovered incidentally [4]. The prevalence of PFO varies depending on the population studied and the imaging techniques used, with racial and ethnic differences reported. For example, in a Japanese autopsy study, the prevalence of PFO in Japanese adults was found to be only 12.5% [17], while another study in an Egyptian population reported a prevalence of 16.3% [18]. These figures are lower than those reported in White populations. These differences warrant further research to clarify the underlying factors contributing to the variability.

The mean diameter of a PFO is typically 4.9 mm, with a range of 1–19 mm. Generally, a PFO does not result in clinically significant blood shunting in terms of hemodynamics [19]. However, under certain conditions, such as when right‐sided cardiac pressure exceeds left‐sided pressure, a PFO may act as a conduit between the right and left atria, leading to an increased RLS. This shunting may allow a thrombus from the venous circulation to bypass pulmonary filtration and enter the arterial circulation, potentially embolizing to the cerebral or other arteries, a phenomenon known as paradoxical embolism.

**FIGURE 1:**
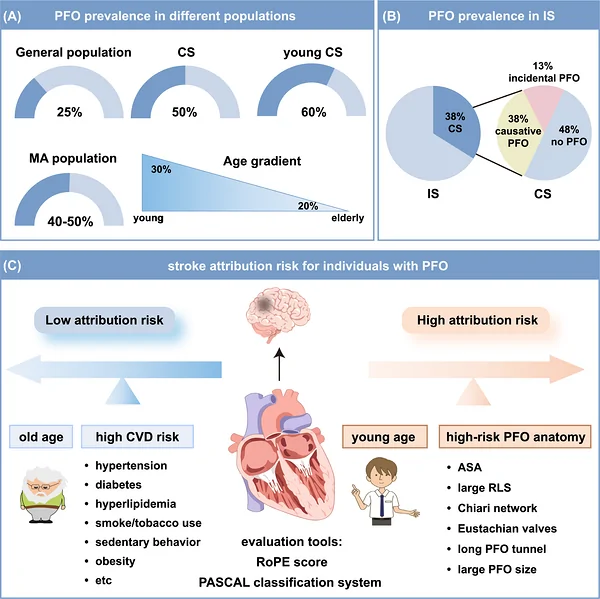
Epidemiological profile of PFO and stroke attribution risk. (A) The prevalence of PFO varies across different populations. It is present in approximately 25% of the general population, although it gradually declines with advancing age, as revealed by autopsy data. In contrast, PFO is detected in nearly 50% of patients with cryptogenic stroke (CS), reaching up to 60% among young patients with CS, and in 40–60% of patients with migraine with aura (MA). (B) Not all PFOs identified in patients with CS are pathogenic. Among all ischemic strokes (IS), approximately 38% are classified as CS, which can be further classified as having causative PFOs (38%), incidental PFOs (13%), or no PFOs (48%). (C) The stroke attribution risk for individuals with PFO depends on both clinical characteristics and PFO morphology. Young age, high‐risk PFO anatomy, and low cardiovascular disease (CVD) risk are associated with a higher probability of PFO‐related stroke, whereas old age and high CVD risk indicate alternative stroke etiologies instead of PFO. The RoPE score and the PASCAL classification system are practical tools for evaluating the causal likelihood of PFO in patients with stroke. Abbreviations: ASA, atrial septal aneurysm; CS, cryptogenic stroke; CVD, cardiovascular disease; IS, ischemic stroke; MA, migraine with aura; PASCAL, PFO‐Associated Stroke Causal Likelihood; RLS, right‐to‐left shunt; RoPE, Risk of Paradoxical Embolism.

Stroke is the second leading cause of death and disability worldwide, accounting for the largest proportion of global disease burden in terms of disability‐adjusted life years, underscoring its substantial public health impact [20]. Ischemic stroke constitutes the majority of cases. Understanding the etiology of ischemic stroke is critical for the development of targeted interventions and public health strategies. While most strokes are attributed to traditional vascular factors such as atherosclerosis, cardiac embolism, or small vessel disease, nearly one‐third of ischemic strokes have no identifiable cause despite extensive diagnostic testing. These strokes are classified as CS and are especially prevalent in younger patients [21]. It is estimated that PFO may contribute to approximately 5% of all ischemic strokes, and up to 10% of strokes in younger individuals [19]. It has been reported that paradoxical brain embolism accounted for 5% of acute ischemic stroke, a result similar to that of PFO contribution [22]. In a Spanish retrospective cohort study of 13,780 patients aged 18–60 years with ischemic stroke, 749 (5.4%) were found to have a PFO [23]. Therefore, PFO has been identified as a potential underlying cause of ischemic stroke, which is also the most common neurological disorder associated with PFO in turn. The epidemiological profile of PFO and stroke attribution risk has been summarized in Figure 1.

## 3 The Risk Factors of PFO

Not all PFOs pose a pathogenic risk or lead to paradoxical embolism and strokes (Figure 1). Therefore, accurately distinguishing between innocent and pathogenic PFOs is crucial. Anatomical variations in PFO structure exist, and the risk of CS varies depending on the morphological type. Data suggest that approximately 5% of the population has particularly dangerous forms of PFO [4, 24]. Identifying those PFOs with a higher risk of stroke is both essential and critical. High‐risk anatomical features of PFO include a larger size, longer tunnel length, ASA, and the presence of an Eustachian valve or Chiari's network [5, 13]. These high‐risk characteristics significantly increase the likelihood of PFO being causally related to stroke. In a study of an Egyptian population, high‐risk anatomical PFOs accounted for 51.5% of all PFOs [18].

### 3.1 ASA and Large RLS

Data have shown that the prevalence of ASA is 2.2% in the general population, and PFO is present in 83% of patients with ASA [25]. In a study of 330 patients with CS and transient ischemic attack (TIA) who underwent PFO closure, 74 (22.4%) were found to have ASA [26]. In another cohort of 154 patients undergoing PFO closure, transesophageal echocardiography (TEE) revealed an ASA prevalence of 58.4% [27]. These findings suggest that the presence of ASA is associated with CS in individuals with PFO.

Moreover, evidence indicates that the grade of RLS is significantly associated with the occurrence of CS [28]. In a study by Gao et al., the RLS grade demonstrated a higher area under the curve in diagnosing CS, compared with RLS type and duration, suggesting that RLS grade may help reduce the misdiagnosis rate, enabling earlier identification of stroke risk [28]. Pu et al. enrolled 113 patients with PFO and CS, and 117 patients with PFO and migraine but without stroke. They found that ASA, Grade III RLS during Valsalva, longer PFO length, and low‐angle PFO, defined as an angle between the inferior vena cava (IVC) and PFO ≤ 10°, were independent risk factors associated with CS [5].

In addition, ASA has been considered an independent risk factor for recurrent stroke in PFO patients, further enhancing the risk of stroke recurrence in those with CS and PFO [29]. Chen et al. analyzed the anatomical characteristics of PFO in 207 patients with CS, revealing that the combination of ASA and a large RLS was associated with recurrent PFO‐related stroke during 2 years of follow‐up. Moreover, PFO patients with ASA had an 11.37‐fold higher risk of recurrent stroke compared with those without ASA, but no independent association was found between RLS and recurrent stroke [29]. Recently, Mas et al. analyzed data from 3740 patients across 6 RCTs and found that patients with both ASA and a large shunt had a significantly lower risk of recurrent stroke after PFO closure compared with those with only a large shunt, a small shunt with ASA, or a small shunt without ASA. The benefit of PFO closure was most pronounced in patients with both a large shunt and ASA at 2 years, with an absolute risk reduction of 5.5%, more than five times greater than the 1.0% reduction seen in the other three groups. It is important to note that a “large shunt” was defined based on the number of bubbles observed on TEE, rather than the directly measured size of the PFO [30]. According to prior findings, the European Stroke Organization Guidelines suggest that patients with CS, ASA, and a large RLS can benefit most from PFO closure [21].

### 3.2 The Size of PFO

In a study by Wang et al., which included 344 patients with PFO, individuals who experienced CS had a significantly larger PFO diameter compared with those without a history of stroke (2.54 ± 0.79 vs. 1.98 ± 1.10 mm), suggesting that PFO diameter may serve as an independent risk factor for CS. Furthermore, PFO diameter demonstrated predictive value for stroke in both younger (<60 years) and older (≥60 years) patients [31]. Similarly, Węglarz et al. analyzed 155 PFO patients with cardiological causes of neurological events and confirmed the predictive value of PFO width for ischemic neurological episodes. Specifically, when the PFO tunnel width was ≥4 mm, the sensitivity and specificity for predicting stroke or TIA were 70 and 55%, respectively [32]. However, several studies have reported inconsistent findings. For instance, Pu et al. found no association between PFO size and the risk of CS [5]. Likewise, Chen et al. reported that a larger PFO was not independently associated with stroke recurrence [29]. This discrepancy may be attributed to several factors, including the retrospective design of these studies, relatively small sample sizes, and the inclusion of a single demographic population (Chinese cohort), all of which could increase the risk of bias in the results.

### 3.3 The Length of the PFO Tunnel

The length of the PFO tunnel has been identified as an independent factor associated with CS [5]. Wang et al. analyzed 134 patients with PFO, either with CS or migraine, and found that the PFO tunnel was significantly longer in the CS group compared with the migraine group (13.4 ± 4.4 vs. 7.8 ± 2.5 mm). In addition, patients with CS had a lower IVC–PFO angle, a higher prevalence of left funnelform morphology, and multiple exits of the tunnel from the left atrium (LA). Multivariate regression analysis indicated that among anatomical PFO features, a longer PFO tunnel, left funnelform morphology, and the presence of multiple left atrial tunnel exits were independently associated with a higher risk of CS. Receiver operating characteristic analysis identified a tunnel length of ≥12 mm, along with left funnelform morphology and multiple left atrial exits, as the optimal cut‐off for diagnosing high‐risk PFO (sensitivity: 92%, specificity: 90%) [33]. However, Chen et al. did not replicate these findings, reporting no association between PFO tunnel length and recurrent ischemic stroke [29]. These discrepancies highlight the need for further prospective studies with larger sample sizes to clarify the role of PFO tunnel length in CS risk.

### 3.4 Chiari Network and Eustachian Valves

Embryonic remnants, such as the Chiari network and Eustachian valves, may increase the risk of paradoxical embolism and CS when associated with a PFO, especially in younger individuals. Evidence indicates that these remnants are frequently observed in patients with PFO and stroke, and their coexistence with a PFO is considered a high‐risk feature [34, 35, 36]. In a study by Trabattoni et al., among 442 patients with PFO, 65.5% presented with CS or TIA, 21.7% with migraine, 10.8% with silent magnetic resonance imaging (MRI) lesions, and 2.0% with decompression sickness (DCS). By analyzing the anatomical characteristics, 20.8% of cases had an ASA, 9.0% had an Eustachian valve, and 19.9% had a Chiari network [37]. A subsequent meta‐analysis of 883 patients revealed that the combined prevalence of Chiari network and Eustachian valves in patients with PFO‐related stroke was 50%, with isolated incidences of 18% for the Chiari network and 43% for Eustachian valves [38]. Ahmed et al. reported the case of a 2‐year‐old boy who presented with sudden‐onset right‐sided hemiparesis and aphasia and was diagnosed with acute ischemic cerebral infarction. A Chiari network was identified in the right atrium, underscoring its potential role in facilitating paradoxical embolism [39]. Wang et al. analyzed 134 patients with CS or migraine and suspected PFO and found that those with CS had a higher incidence of Eustachian valves or Chiari network compared with migraine patients (14.3 vs. 3.5%) [33]. These findings highlight that the presence of the Chiari network and Eustachian valves may play a role in the development of PFO‐mediated paradoxical embolism and CS. However, the contribution of these embryonic remnants to PFO‐mediated paradoxical embolism is still underrecognized. There remains a lack of research addressing their role in clinical decision‐making, particularly regarding PFO closure and the selection of antithrombotic therapy.

### 3.5 Other Risk Factors for CS

In a case–control study by Putaala et al., risk factors for young‐onset ischemic CS were evaluated. The study found that nontraditional risk factors played a more prominent role in the development of ischemic CS when PFO was present, particularly in patients aged 18–39 years, compared with those aged 40–49 years. Additionally, migraine with aura was identified as the greatest contributor to ischemic CS in female patients with PFO, with a population‐attributable risk as high as 45.8% [40].

Interestingly, Kalkan et al. reported that electrocardiography (ECG) could distinguish between high‐risk and low‐risk PFO anatomies. In a retrospective single‐center study, high‐risk patients, as assessed by the Nakayama risk score, were more likely to present with crochetage R waves, which were strongly associated with large RLS and high‐risk PFO anatomy. These findings suggest that anatomical risk features and the magnitude of the shunt can drive ECG changes, particularly the crochetage R wave, which could facilitate noninvasive risk stratification in CS [41].

Qi and colleagues assessed the prognostic value of left atrial strain using two‐dimensional (2D) speckle tracking imaging to predict the risk of CS related to PFO and RLS. The study found that patients with PFO and RLS had impaired left atrial function. Left atrial reservoir strain and left atrial conduit strain were identified as independent predictors of CS risk related to PFO and RLS, demonstrating strong predictive performance. These findings suggest that these measures could serve as efficient predictors and indicators of CS in patients with PFO and RLS [42].

Furthermore, there may be an association between certain trigger factors and ischemic stroke in young individuals with PFO. Immens and colleagues indicated that flu‐like disease, fever, and vigorous exercise could convert an asymptomatic PFO into a stroke‐causing PFO in the young population. However, this study used a questionnaire format, and any research relying on patient recall may be prone to recall bias, potentially affecting the reliability of its conclusions. Further investigation is required to elucidate the mechanisms by which a PFO shifts from being a benign entity to one that poses a risk of harm [43]. Identifying high‐ or low‐risk PFO individuals and then selecting patients who are likely to benefit from interventional device closure remains a key challenge. Therefore, whether to establish a risk stratification system based on the anatomical features of PFO is worth considering.

## 4 The Pathophysiology of PFO

### 4.1 Genetic Predisposition of PFO

PFO has been considered to have a genetic predisposition [44]. Therefore, early identification of the PFO‐related mutant genes may help determine the risk of developing stroke or TIA, enabling timely clinical intervention and reducing the occurrence of ischemic cerebrovascular events (CVEs). Several studies have aimed to elucidate the pathogenic gene in PFO. Recently, a Chinese research team performed a genome‐wide association study to evaluate common genetic variants associated with PFO. They found that among the assessed variants, rs1227675732, rs62206790, rs879176184, and rs13115019 were associated with a high risk of PFO, whereas rs57922961 was protective [45]. Uncovering susceptibility loci for PFO may help us to elucidate its pathogenesis. The X‐linked *NONO* gene, involved in transcriptional regulation, RNA splicing, and DNA repair, has been reported to be associated with PFO due to its pathogenic variants [46]. Li et al. conducted whole‐exome sequencing on 25 patients with PFO and revealed pathogenic mutations in LDLR, SDHC, and NKX2‐5 genes, suggesting their role in PFO development. Among them, the NKX2‐5 gene may play a critical role by interacting with signaling pathways that regulate cardiomyocyte characteristics [44]. The genetic findings regarding PFO may provide potential therapeutic targets for future research.

### 4.2 Paradoxical Embolism and In Situ Thrombus

**FIGURE 2:**
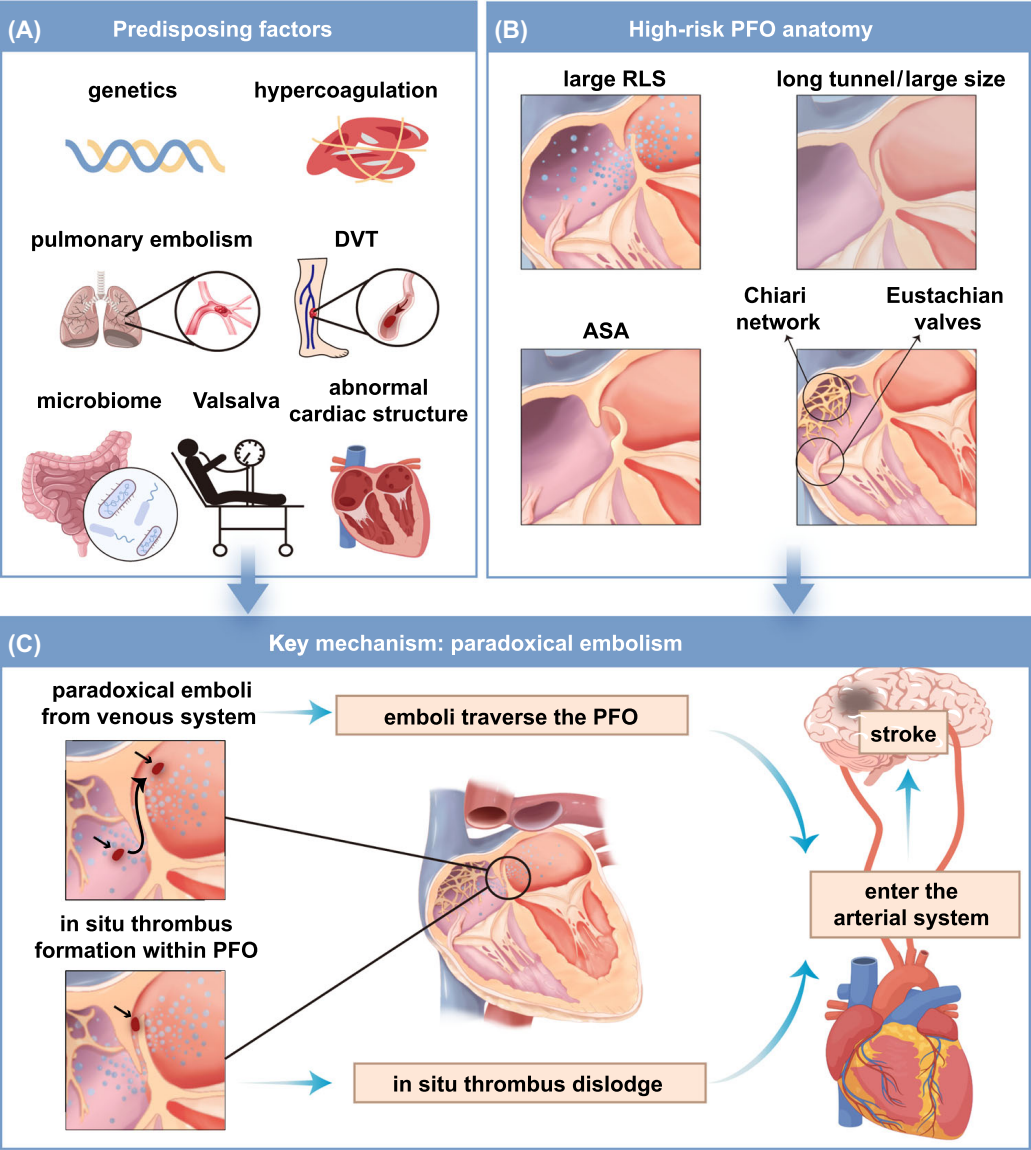
Pathophysiological mechanisms of PFO‐related stroke. The key mechanism underlying PFO‐related stroke is mediated by paradoxical embolism. (A) Multiple predisposing factors, including genetic susceptibility, hypercoagulation, pulmonary embolism, deep vein thrombosis (DVT), microbiome alterations, Valsalva maneuver, and abnormal cardiac structure, may increase thromboembolic risk and augment right‐to‐left shunt (RLS). (B) High‐risk PFO anatomies, including large RLS, a long tunnel, a large size, atrial septal aneurysm (ASA), Chiari network, and Eustachian valves, may facilitate in situ thrombus formation and promote emboli transit. (C) The paradoxical emboli from the venous system can traverse the PFO and enter the arterial system, while in situ thrombi formed within the PFO tunnel may also dislodge and contribute to arterial embolism, ultimately resulting in ischemic stroke. Abbreviationss: ASA, atrial septal aneurysm; DVT, deep vein thrombosis; RLS, right‐to‐left shunt.

An opening PFO can serve as a conduit, allowing microemboli originating in the venous system to traverse it and enter the arterial system. These paradoxical emboli can block branches of the cerebral arteries, leading to ischemic stroke. PFO‐related stroke is a complex pathophysiological process, and paradoxical embolism has been believed to be the main mechanism underlying PFO‐associated stroke (Figure 2). Several studies have observed a thrombus straddling a PFO using TEE, providing direct evidence for the mechanism of paradoxical embolism [47]. Deep vein thrombosis (DVT) is regarded as a source of emboli in PFO. In one study of 217 patients with stroke or TIA and concomitant PFO who underwent compression ultrasonography, DVT was detected in 7.8% of the cohort [48].

PFO itself may be an etiopathogenetic contributor of the embolic source. Thrombus can form within the PFO tunnel, and in situ thrombus formation has been recognized as a putative mechanism of PFO‐related stroke [49]. These microthrombi within PFO may dislodge, travel into the LA and systemic circulation, and subsequently cause embolism of target organs (Figure 2). Yan et al. enrolled 117 patients with PFO and without known vascular risk factors, 43 presenting with stroke or TIA, 49 with migraine, and 25 with no symptoms. Using optical coherence tomography (OCT), the study found in situ thrombi in 36 (83.7%) stroke patients, 28 (57.1%) migraine patients, and none of the asymptomatic patients, indicating a higher prevalence of in situ thrombus in stroke patients. Moreover, in situ thrombus in PFO was strongly associated with stroke risk and may serve as a valuable predictor [50]. Recently, a case of a 43‐year‐old man who suffered cerebral infarction attributed to PFO was reported. OCT examination confirmed that the emboli originated from the PFO. The patient received PFO closure and experienced no recurrence of cerebral infarction at the 6‐month follow‐up [51].

In addition to venous thrombosis and in situ thrombus within PFO, recent studies have found that in situ thrombus in the right atrium may also serve as a source of paradoxical embolism mediated by PFO. Zhu and colleagues documented that the presence of PFO can induce the formation of in situ microthrombus on the right atrial septum, which in turn increases the risk of PFO‐associated stroke [52].

### 4.3 Additional Pathogenesis Involved in PFO‐Associated Stroke

Several studies have reported that the microbiome may be involved in the development of PFO‐associated stroke. Manzoor et al. analyzed the oral microbiome and showed that the combination of oral dysbiosis and high‐risk PFO may constitute a critical but underappreciated factor in the etiology of ischemic CS [53]. However, given the limited available research, it is insufficient to draw definitive conclusions. Further studies are needed to elucidate the precise mechanisms of these interactions and to develop targeted intervention strategies.

Recently, endothelial dysfunction has been reported to be associated with PFO. In a study by Tworek et al., the levels of serum serotonin, a marker of endothelial dysfunction, were higher in patients with PFO‐related CS, compared with healthy individuals. However, no correlation was found between serotonin levels and PFO anatomical characteristics, such as length and width [54]. PFO‐related CS may result from multiple mechanisms acting together. Evidence indicates that there may be an association between abnormal atrial structure and the pathogenesis of PFO‐related stroke, as they can result in abnormal cardiac structure and accumulation of circulating biomarkers, potentially contributing to vascular endothelial injury and thrombosis [55].

## 5 The Clinical Features of PFO

Although the majority of PFOs are benign and asymptomatic, they can present with a variety of clinical manifestations, including cerebral disorders (such as CS, TIA, and migraine) and other systemic embolic events, which may lead to serious clinical consequences. Identifying these diverse manifestations is important for implementing early and appropriate management strategies, thereby reducing the morbidity burden associated with PFO.

### 5.1 PFO and Ischemic Cerebral Diseases

#### 5.1.1 PFO and Ischemic Stroke

Identifying the etiology of stroke is essential to guide effective treatment. CS accounts for one‐fourth of acute ischemic strokes, with most cases attributed to embolic etiologies [56]. The increasing incidence of young‐onset ischemic stroke is largely driven by a rise in CS [40]. Among the various organs affected by PFO, the brain is the most common target, and substantial evidence has established a strong association between PFO and CS. A retrospective study found that 17.2% of individuals with PFO had experienced a CS [31]. Compared with ischemic stroke patients (aged 18–60 years) without PFO, those with PFO tend to be younger, have fewer risk factors, and exhibit lower all‐cause mortality [23]. In turn, PFO is particularly prevalent in younger patients with CS. Epidemiological studies indicate that nearly half of patients aged 60 years or younger with CS have a PFO [19]. In clinical practice, PFO has therefore gained increasing recognition as a potential etiological factor in young individuals with unexplained stroke. Previous studies have suggested that approximately 60% of CS cases in young adults may be attributable to PFO [57]. In addition, the prevalence of PFO is significantly higher in patients with CS than in those with an established stroke etiology [23].

However, not all PFOs are pathogenic. The distinction between causative and incidental PFO is particularly important in stroke evaluation. An epidemiologic study indicated that among patients younger than 55 years with ischemic CS, 38% had causative PFOs, 13% had incidental PFOs, and 48% had no PFO. These findings suggest that PFO accounts for a substantial proportion of ischemic strokes and may represent a more common embolic source than low‐burden atrial fibrillation (AF) [25].

Interestingly, beyond epidemiological associations, patients with PFO‐related CS may also exhibit distinct clinical and biological characteristics. Several biomarkers have been proposed to help identify PFO‐associated stroke. Among the biomarkers of cardiac fibrosis, remodeling, and inflammation, low levels of galectin and osteoprotegerin were independently associated with PFO‐related stroke, suggesting that the two biomarkers may assist in identifying patients with PFO‐related strokes [58]. As rises in serum galectin and osteoprotegerin levels are involved in a higher risk of cardiovascular events, the low levels in patients with PFO‐related strokes may reflect the low cardiovascular burden. In addition, the triglyceride glucose (TyG) index, considered a marker of insulin resistance, has emerged as another potential biomarker. Ayca et al. retrospectively enrolled 129 patients with PFO who experienced embolic stroke and 108 subjects with PFO but without stroke. An elevated TyG index was observed in the PFO with stroke group, which showed the highest sensitivity in predicting stroke compared with triglyceride or glucose levels alone. The researchers believed that assessing the TyG index may help optimize the management of CS patients with PFO [59]. Another retrospective study by Esin et al. confirmed the conclusion by demonstrating that patients with CS and PFO have higher TyG index levels, which could serve as an independent predictor of CS in the PFO population [60]. Therefore, the TyG index may be considered a critical biomarker for identifying CS risk in this population.

#### 5.1.2 PFO and Migraine

Migraine is the second most prevalent neurological disorder and shares overlapping pathophysiological mechanisms and risk factors with ischemic stroke [61, 62, 63]. Moreover, patients with migraine consistently show an increased prevalence of vascular brain lesions on neuroimaging and a heightened risk of ischemic stroke [34]. Despite extensive research, the pathophysiology of migraine remains incompletely understood. Accumulating evidence suggests a positive causal association between migraine and PFO, with the presence of PFO potentially increasing the risk of migraine [61, 63, 64]. Compared with healthy individuals, PFO is more prevalent in patients with migraine, with a prevalence of 40–60% [34, 64, 65]. Meanwhile, compared with migraine patients without PFO, those with PFO tend to experience earlier symptom onset, have a higher rate of migraine family history, and claim greater headache severity [66].

Migraines are broadly classified into migraine with aura and migraine without aura, with approximately 30% of patients experiencing aura [63]. A community‐based cross‐sectional study conducted in China screened 881 individuals with PFO from a cohort of 3741 residents over 20 years of age and found that the prevalence of migraine without aura was significantly higher in individuals with PFO than in those without (12.83 vs. 7.83%), suggesting that PFO may increase the morbidity risk of migraine without aura [65]. However, growing evidence indicates that the association between PFO and migraine with aura may be even stronger [64]. Patients with migraine and PFO consistently exhibit a higher prevalence of aura [66]. A meta‐analysis of 27 studies involving 8875 patients demonstrated that the PFO prevalence was particularly elevated in migraine with aura compared with migraine without aura, indicating a closer relationship between PFO and migraine with aura [64]. Recently, Che et al. conducted a Mendelian randomization study using aggregated data from a Finnish database and found that PFO was significantly associated not only with overall migraine, but also with both migraine with aura and migraine without aura [63].

Beyond prevalence, increasing attention has focused on the relationship between RLS grade and migraine severity. In a study by Gu et al., 190 migraine patients were stratified into Grades 0–III based on the number of microbubbles detected in the LA after right atrial opacification on contrast transthoracic echocardiography (cTTE). Patients with higher microbubble grades had markedly elevated Migraine Disability Assessment (MIDAS) and Headache Impact Test‐6 (HIT‐6) scores, demonstrating a clear dose–effect relationship between shunt magnitude and migraine severity [67]. These findings provide direct evidence that RLS contributes to migraine.

Several mechanisms have been proposed to explain the link between PFO and migraine. One possible hypothesis is that vasoactive substances from the venous circulation may bypass pulmonary filtration through a PFO and directly enter the cerebral circulation, thereby triggering migraine attacks in susceptible individuals [34]. Supporting this concept, Dong et al. analyzed 371 migraine patients and found that PFO was independently associated with aura, lower cystatin C levels, and lower calcium concentrations, suggesting that these factors may serve as biomarkers in migraine patients with PFO. Plasma metabolomics in patients before and after PFO closure revealed that 5‐hydroxytryptamine (5‐HT) and glutathione (GSH) metabolites were differentially expressed. Combined with in vivo experiments using PFO‐susceptible mice, the authors proposed that PFO may influence the posterior region of the brain by regulating 5‐HT and GSH. Their findings suggest that PFO closure may alleviate migraine through improved clearance of vasoactive substances and alleviation of redox signaling [68].

Another important proposed hypothesis is the microembolus theory. According to this hypothesis, paradoxical microemboli from the venous system may pass through a PFO into the cerebral circulation, leading to cerebral hypoperfusion and cortical spreading depression, which may subsequently trigger migraine attacks [61, 63]. In addition, inflammation and oxidative stress may further contribute to migraine pathogenesis in this population. Recently, Tang et al. investigated the neural basis of the relationship between migraine and PFO using independent component analysis of functional MRI data and found that patients with both migraine and PFO exhibited widespread functional connectivity alterations within and across brain networks. These findings may help further elucidate the complex neurobiological basis of PFO‐related migraine [69].

Biomarker research has also provided additional insights. Previous studies have shown that certain metabolites change significantly before and after PFO closure in migraine patients, suggesting potential diagnostic value. Among these, linoleic acid showed strong diagnostic potential and may serve as a biomarker among this population [70]. Li et al. further reported that migraine patients with PFO had higher serum calcitonin gene‐related peptide (CGRP) levels than those without PFO, indicating that CGRP may be an independent risk factor for migraine associated with PFO. Moreover, CGRP levels increased in parallel with RLS grade, suggesting that elevated serum CGRP may have predictive value for identifying PFO in migraine patients [71].

Despite numerous studies, the role of PFO in migraine remains controversial because direct mechanistic evidence from experimental and clinical studies is still limited. Defining migraine specifically attributable to PFO remains challenging, and it is more possible that PFO and migraine coexist, rather than exhibiting a direct causal relationship. At present, routine screening for PFO in all migraine patients is not considered necessary.

#### 5.1.3 PFO and Epilepsy

Epilepsy is a common chronic neurological disorder characterized by recurrent unprovoked seizures. PFO is a common comorbidity in patients with epilepsy, and emerging evidence suggests a potential association between the two conditions [72]. In a Chinese cohort study, the incidence of epilepsy in patients with PFO was 8.58 per 1000 person‐years, which was higher than that observed in patients with atrial septal defect (ASD). The study further demonstrated that individuals with PFO had a significantly higher risk of epilepsy than those with other types of congenital heart disease. Notably, this elevated risk was reduced in patients who underwent PFO closure, suggesting a possible contributory role of PFO in epilepsy development [73]. Clinical case reports have also raised interest in the therapeutic implications of PFO closure for epilepsy management. In a small pilot study, Ji et al. enrolled 31 epilepsy patients with PFO and, for the first time, demonstrated that PFO closure significantly reduced seizure frequency, duration, and severity [74]. Their findings suggested that patients with drug‐resistant epilepsy who also have PFO with a large shunt may be ideal candidates for closure intervention.

Despite these observations, the shared etiology and pathophysiological mechanisms linking PFO and epilepsy remain poorly understood. Recently, Zhang et al. have provided important mechanistic findings. Using a poly(I:C)‐induced severe maternal immune activation (MIA) rat model, the investigators demonstrated that a single MIA insult could simultaneously increase both seizure susceptibility and PFO prevalence in offspring, suggesting that MIA may represent a common upstream etiological factor for both conditions. Integrated transcriptomic and snRNA‐seq analyses revealed that activation of the Hippo signaling pathway, coupled with downregulation of Yap1, served as a shared mechanism disrupting both neurodevelopment and cardiac development, thereby contributing to this comorbidity [72].

### 5.2 PFO and Systemic Embolism

While CS remains the most common concern associated with PFO, PFO‐mediated paradoxical embolism is also an important cause of systemic arterial embolism, affecting the retinal, coronary, visceral, and peripheral arteries. However, compared with CS, systemic embolism caused by PFO is less frequent.

#### 5.2.1 Coronary Embolization and Myocardial Infarction

Although the most common manifestations of PFO are neurological symptoms, PFO is associated with other clinical conditions, such as coronary embolism and acute myocardial infarction (AMI). In recent years, growing evidence has suggested a potential association between PFO and major adverse cardiovascular events (MACEs) in patients with coronary artery disease (CAD).

Li et al. retrospectively analyzed 2667 patients with suspected CAD and found that, during a median follow‐up of 86 months, patients with PFO had a higher incidence of MACEs, which were defined as cardiac death, nonfatal myocardial infarction (MI), and ischemic stroke, than those without PFO. Multivariate analysis revealed that PFO is an independent predictor of MACE in CAD patients. These findings were the first to indicate that PFO is associated with adverse long‐term prognosis in CAD patients [75]. Beyond its prognostic significance, PFO may directly contribute to coronary events through paradoxical embolism. Coronary artery embolism accounts for approximately 3% of MI cases involving nonobstructive coronary arteries, and AMI caused by PFO‐mediated paradoxical embolism has been described in multiple case reports [76, 77, 78]. In such cases, nonatherosclerotic etiologies should be considered, especially in isolated distal culprit coronary lesions [77]. Considering the substantial global burden of AMI, PFO‐related paradoxical coronary embolism deserves greater clinical attention. Nevertheless, the clinical significance of PFO in patients with CAD and AMI remains underrecognized currently.

#### 5.2.2 PFO and Acute Pulmonary Embolism

Acute pulmonary embolism (PE) is a potentially life‐threatening disease that usually presents with hypoxia. PFO is a common condition in patients with PE [79], with one study enrolling 129 PE patients reporting a prevalence of 36.7% [80]. Patients with PE who also have a PFO often present with more severe clinical manifestations and may require more aggressive therapeutic interventions, such as catheter‐directed thrombolysis [79, 81, 82]. Moreover, increasing evidence has shown that the presence of PFO in PE patients carries an increased risk of ischemic stroke and mortality compared with PE patients without PFO [79]. Nykl et al. also demonstrated that PE patients with a PFO had a higher prevalence of acute and subacute ischemic brain lesions on MRI, highlighting the potential neurological consequences of this combination [80]. However, the current evidence remains inconsistent. One previous study did not find that PFO increased the in‐hospital mortality among patients with PE [81]. Despite these discrepancies, the presence of refractory hypoxia in acute PE should raise clinical suspicion for PFO [82].

#### 5.2.3 PFO and Other Artery Occlusion

In theory, once paradoxical emboli enter the arterial circulation through a PFO, they may travel to virtually any organ and cause embolic events in diverse arteries. Beyond CS and AMI, PFO‐mediated paradoxical embolism has also been implicated in retinal artery occlusion (RAO), visceral and peripheral arterial embolism. However, the available literature on extracerebral paradoxical embolism is primarily limited to case reports.

RAO is an ophthalmic emergency characterized by sudden visual loss, and paradoxical emboli represent a rare but potentially underrecognized etiology. Several reports have highlighted this association. Tanaka et al. reported a 49‐year‐old Japanese man diagnosed with central RAO whose MRI revealed an acute ischemic lesion in the right occipital subcortex. After extensive etiological evaluation, PFO was identified as the cause of both retinal and cerebral ischemia [83]. In addition, Shi et al. described a 35‐year‐old male who presented with RAO attributable to PFO, in whom OCT detected multiple white thrombi within the tunnel of the PFO [84]. Based on prior studies, Fekri et al. summarized 23 cases of PFO‐associated RAO and found that young age, migraine, recent immobility, vigorous exercise, pregnancy, and the absence of known cardiovascular risk factors may serve as indicators pointing toward paradoxical embolism as the underlying cause [85]. These findings suggest that clinicians should be alert to the presence of PFO in young patients with RAO, particularly in those without an identifiable cause. In a retrospective case series enrolling 18 young patients aged under 45 years with RAO and no traditional cardiovascular risk factors, PFO was identified as the presumed cause in 17% of cases. None of these patients underwent PFO closure, and no recurrent stroke or RAO occurred during a mean follow‐up of 3.6 ± 3.2 years. These findings suggest that the long‐term risk of recurrent ocular or CVEs may be relatively low when no other risk factor coexists with PFO. However, further validation is needed in large‐scale studies [86].

PFO‐associated paradoxical embolism has also been reported as a cause of visceral arterial occlusion. Rashwan et al. reported a 30‐year‐old female with splenic infarction due to PFO‐associated paradoxical embolism [87]. Similarly, Annan et al. reported a 68‐year‐old woman presenting with sudden severe abdominal pain and vomiting who was diagnosed with acute superior mesenteric artery occlusion by computed tomography angiography. PFO‐mediated paradoxical embolism was presumed to be the most plausible etiology [88]. These reports emphasize that patients with venous thrombosis in combination with unexplained visceral embolism may warrant evaluation for PFO.

Several reports have illustrated that PFO‐associated paradoxical embolism can obstruct medium‐ or large‐peripheral arteries, causing acute limb ischemia, as well as small vessels resulting in thrombotic vasculopathy [89, 90, 91]. For example, a 66‐year‐old man experienced occlusions of the left dorsalis pedis and distal posterior tibial arteries, in the setting of bilateral popliteal DVT and a PFO. In this patient, acute limb ischemia was attributed to paradoxical embolism secondary to the PFO because no other alternative etiology was found. He subsequently underwent PFO closure and surgical embolectomy [90]. Similarly, Kwan et al. reported a 20‐year‐old man who presented with painful dusky digits of both feet and was diagnosed with PFO‐related acute thrombotic vasculopathy causing cutaneous ischemia [89]. Evidence indicates that acute limb ischemia caused by PFO‐mediated paradoxical embolism is often accompanied by DVT, PE, or concomitant cerebral or visceral ischemia [91]. Elzawy et al. reported a 29‐year‐old woman with left popliteal vein thrombosis, submassive PE, and concurrent acute limb ischemia caused by PFO‐mediated paradoxical embolism [91]. The authors proposed that PFO should be suspected in young patients with acute limb ischemia who have ipsilateral DVT.

Although rare, these cases illustrate that PFO‐mediated paradoxical embolism can involve any artery in the body. Therefore, in patients with unexplained arterial occlusion, clinicians should remain vigilant for the possibility of PFO. Nevertheless, diagnosing PFO‐mediated visceral and peripheral embolism remains challenging, mainly due to its nonspecific presentation [91]. Early recognition of PFO‐mediated embolism is essential, as timely intervention may reduce complications, disability, and mortality, especially when systemic arteries are affected.

### 5.3 PFO and DCS

DCS is one of the most serious medical risks in scuba diving, caused by the compression and decompression of gases as depth changes. Neurological manifestations, including stroke or TIA, are the common symptoms of DCS. Despite the growing popularity of scuba and freediving, DCS remains incompletely understood. Increasing evidence suggests that PFO may play an important role in DCS, illustrated by several case reports describing DCS secondary to PFO. Fernandez‐Cisneros et al. reported a 55‐year‐old man who was diagnosed with severe Type II DCS, which may be caused by PFO confirmed by echocardiography [92]. Another report described a 28‐year‐old woman who developed DCS when performing a scuba air dive, with constitutional and neurological symptoms, including multifocal cerebral embolic infarcts. A PFO and severe right ventricular (RV) dilation were detected in this patient, and the authors proposed that provocative decompression led to venous bubble overload, increasing pulmonary artery pressure and causing RV dilatation; this facilitated RLS of bubbles across the PFO, resulting in significant neurological deficits [93].

Given these clinical observations, professional statements by the South Pacific Underwater Medicine Society and the United Kingdom Diving Medical Committee recommend considering evaluation for RLS or in selected high‐risk individuals rather than universal screening. These cohorts include a history of cerebral, spinal, vestibulocochlear, cardiovascular, or cutaneous DCS; history of migraine with aura; history of CS; first‐degree relative with PFO or ASD; history of congenital heart disease. A spontaneous RLS at rest or a large shunt that is provoked by maneuvers is considered a definite risk factor for DCS with these specific conditions [94]. Similarly, current guidelines do not support routine PFO closure solely for primary prevention of DCS in individuals without PFO‐related stroke [95]. In addition, the data about the long‐term outcomes of patients who continue to dive following PFO closure remain scarce. Therefore, it is expected to perform an RCT to evaluate the efficiency and safety of PFO closure to reduce recurrent DCS risk in divers with PFO.

### 5.4 PFO and Hypoxemic Disorders

Hypoxemia attributable solely to PFO is rare and has been described only sporadically in isolated case reports. Allaham et al. presented a rare case of an 80‐year‐old male patient with progressive dyspnea and refractory hypoxemia due to PFO‐mediated RLS. After PFO closure, the patient had a complete resolution of his hypoxia within 24 h [96]. Witte et al. reported a 73‐year‐old woman with progressive dyspnea on exertion. After PFO closure, her resting SaO_2_ improved from 87% preclosure to 98% [97]. In patients with certain comorbidities, the existence of a PFO further exacerbates the occurrence of systemic hypoxemia, serving as an amplifying factor, and has been reported to be associated with several hypoxemic disorders, including platypnea–orthodeoxia syndrome (POS), chronic obstructive pulmonary disease (COPD) and obstructive sleep apnea (OSA).

#### 5.4.1 PFO and POS

POS is an uncommon clinical disorder characterized by positional dyspnea and hypoxemia, with symptoms worsening in the upright position and relief when supine. The pathophysiology of POS remains incompletely understood, but evidence indicates that PFO is a commonly encountered structural anomaly in POS, which is commonly associated with PFO‐mediated RLS‐induced hypoxemia [98]. However, the prevalence of POS in individuals with PFO remains low, mainly linked to complex interatrial septal anatomy, including a higher prevalence of septal aneurysms and large PFO size [99]. The size of the PFO shunt is particularly critical, with larger shunts posing a greater risk of significant hypoxia in POS patients [100]. PFO closure has consistently been associated with significant improvements in hypoxemic symptoms and oxygen saturation in most reported cases [98, 101]. Castillo‐Rodriguez et al. reported a 32‐year‐old woman who had progressive dyspnea and a decline in SpO_2_ of more than 10% when seated versus when supine. A PFO was diagnosed, and the patient underwent PFO closure, reaching complete resolution of both dyspnea and hypoxemia [102]. Similarly, a man in his mid‐70s with POS experienced complete resolution of breathlessness or hypoxemia in any position or at any exertional level after successful PFO closure [103]. In a single‐center Spanish study of 11 patients with POS, the median oxygen saturation improved from 83% before the PFO closure to 97% afterward [104]. Another Italian retrospective multicenter study enrolled 48 patients with POS and demonstrated significant improvements in orthostatic arterial oxygen in all patients after PFO closure, with an average increase of 8.65% from baseline, and sustained symptomatic relief through the 6‐month follow‐up [99].

Given these findings, screening for PFO should be considered in patients with unexplained POS. Moreover, current evidence suggests that PFO closure appears to be an effective and safe therapeutic strategy, offering significant symptomatic and physiological benefits for this population. To support this theory, the Society for Cardiovascular Angiography & Interventions (SCAI) guidelines have recommended PFO closure in selected POS patients [95].

#### 5.4.2 PFO and COPD

In theory, COPD may increase right atrial pressure, thereby exaggerating RLS across a PFO [101]. Recently, Balakumar et al. conducted a meta‐analysis to evaluate the association between PFO and COPD. Based on six cross‐sectional studies, COPD patients had nearly three times higher odds of having a PFO than controls. Importantly, compared with COPD patients without a PFO, those with a PFO had worse outcomes, including lower oxygen saturation, reduced arterial oxygen partial pressure, and higher pulmonary arterial pressure [105]. Further studies are warranted to evaluate the long‐term impact of PFO on prognosis in patients with COPD, and to determine whether screening for PFO is necessary in high‐risk COPD patients. However, to date, no available evidence has supported a clear benefit of PFO closure for improving hypoxemia in COPD patients.

#### 5.4.3 PFO and OSA

OSA is a common sleep‐related breathing disorder often accompanied by oxygen desaturation of varying degrees [106]. Emerging evidence suggests a potential association between OSA and PFO. Lau et al. illustrated a prevalence of 47.1% of PFO in 102 patients with OSA, compared with an incidence of 26.0% in healthy controls, suggesting a higher prevalence of PFO in patients with OSA [106]. PFO contributes to the increased severity of intermittent hypoxia in patients with moderate to severe OSA [107]. Compared with patients who have a PFO alone, those with both PFO and OSA have a greater prevalence of RLS, which may further exaggerate the burden of hypoxia and the risk of paradoxical thromboembolism [108]. Prior studies showed that in OSA patients with a PFO, oxygen desaturation occurs more frequently and is directly proportional to the frequency of respiratory disturbances, compared with those without a PFO [109]. In addition, patients with severe OSA already have an increased risk of stroke, and PFO further reinforces this association. Schwarz et al. reported four cases of OSA patients who developed stroke, which was presumed to be related to the presence of PFO. Following PFO closure and continuous positive airway pressure therapy for OSA, none of the patients experienced recurrent stroke during follow‐up [108].

However, the available evidence about the role of PFO in OSA remains limited and debated. Lau et al. found no significant effect of PFO on the severity of nocturnal oxygen desaturation [106]. Similarly, in a study by Lv and colleagues, 509 patients with OSA were divided into two groups based on the presence or absence of PFO. Although PFO exacerbates intermittent hypoxia in those with moderate‐severe OSA, the presence of PFO did not affect minimum oxygen saturation or oxygen desaturation index compared with those without PFO [107]. These inconsistencies highlight the need for further investigation to explore the relationship between OSA and PFO. In addition, OSA is associated with a prothrombotic state, characterized by an increased risk of thrombus formation and stroke [101]. In this context, whether the coexistence of PFO exerts a synergistic or a causal effect on stroke risk in OSA patients also warrants further study.

#### 5.4.4 PFO and Hypoxemia Under Other Conditions

Acute mountain sickness (AMS) often occurs in individuals who travel to high altitudes and is associated with a decrease in partial pressure of oxygen, leading to tissue hypoxia [110]. Evidence suggests that PFO may be a risk factor for AMS. West et al. enrolled 137 hikers and found that the prevalence of PFO was higher in hikers with AMS than in those without AMS (63 vs. 39%), with the presence of PFO significantly increasing the risk of developing AMS in hikers [111]. The possible mechanism involves PFO exacerbating hypoxemia through RLS, thereby contributing to the onset of AMS [110]. In addition, cystic fibrosis is a multisystemic, autosomal recessive disease. Belt et al. found that PFO was more prevalent among cystic fibrosis patients with hypoxemia. In these patients, the presence of a PFO is associated with hypoxemia at higher FEV_1_% levels and with increased pulmonary exacerbations; however, no such association is observed with overall survival or lung transplantation [112].

When combined with RV failure, PFO may cause severe hypoxemia. A 40‐year‐old man with arrhythmogenic RV dysplasia and advanced RV failure developed refractory hypoxemia due to a large PFO, even on mechanical circulatory support. After PFO closure, oxygenation and RV function were improved, allowing discontinuation of mechanical circulatory support [113]. These observations suggest that PFO closure may be feasible in RV failure patients requiring mechanical circulatory support. Therefore, PFO closure combined with effective treatment of the underlying disease may be beneficial in improving hypoxemia.

Although some patients have shown improvements in hypoxemia and related symptoms after percutaneous PFO closure, these studies were based on small case series or individual cases, with limited sample sizes and long‐term follow‐up. While case reports can provide important clinical insights, particularly offering new perspectives and possibilities for managing complex, high‐risk situations, they cannot be directly extrapolated to serve as the basis for general clinical practice. Moreover, hypoxemia is often caused by multiple factors, making it difficult to determine the specific contribution of PFO relative to underlying pulmonary disease. Due to limited data, no relevant guidelines currently exist to guide the management of these disorders. Current evidence does not support PFO closure as a routine treatment for hypoxemia associated with COPD or OSA. It may only be considered after individualized decision‐making in a very small subset of patients whose degree of hypoxemia is clearly disproportionate to the severity of their lung disease and in whom rigorous evaluation suggests that PFO is a major contributing factor.

## 6 The Diagnosis of PFO

Screening for PFO in otherwise healthy and asymptomatic individuals shows limited clinical value. According to the British Cardiovascular Intervention Society, PFO screening is recommended in the following high‐risk populations, including patients younger than 60 years with unexplained stroke, TIA, MI, or other systemic embolism; patients older than 60 years with recurrent unexplained stroke, TIA, MI, or systemic embolism; divers presenting with certain types of DCS; and individuals with unexplained hypoxic syndromes [114]. For patients in whom PFO is highly suspected to be causative, accurate detection constitutes a critical first step for subsequent etiological attribution and therapeutic decision‐making. Enhancing PFO diagnostic testing in these patients is expected to lead to better health outcomes and cost savings. Studies have shown that in a simulated cohort of 1000 patients, raising the PFO diagnostic testing rate from 54 to 100% is projected to avert 63 recurrent strokes, yielding 23 life‐years saved and 286 quality‐adjusted life years gained [115].

### 6.1 Contrast Transcranial Doppler Ultrasound

Currently, the commonly used methods for diagnosing PFO in clinical practice primarily include contrast transcranial Doppler ultrasound (cTCD), transthoracic echocardiography (TTE)/cTTE, and TEE/contrast TEE (cTEE), each offering distinct advantages in detection. Among these, cTCD is a noninvasive and easily accessible technique for detecting microbubbles within the cerebral circulation, which has been recommended as a first‐line screening tool for detecting RLS and PFO in multiple guidelines and expert consensus statements due to its high selectivity [21, 116]. Evidence has shown that the sensitivity of cTCD for detecting PFO is higher than that of cTTE and comparable to that of cTEE, with a sensitivity of 0.91 for cTCD and 0.86 for cTTE, respectively [117, 118]. In a study by Khattab et al., the diagnostic concordance rate between cTCD and TEE for detecting RLS and PFO reached 91.5% [119]. However, the specificity of cTCD for diagnosing PFO‐RLS is relatively low at 0.87 [118], and an important limitation is that it cannot distinguish between intracardiac and extracardiac shunting or provide detailed morphological structure information about PFO [120].

Although the Valsalva maneuver remains the standard method during cTCD examination, its execution relies heavily on patient cooperation, which limits its applicability in special populations such as children and weak patients. Consequently, nonstandard maneuvers may reduce diagnostic accuracy. To address this limitation, several modified maneuvers and techniques have been explored. Recently, Tian and colleagues introduced a modified Valsalva blowing device, which was evaluated in a single‐center study involving 805 patients with suspected PFO. These patients underwent cTCD using both the traditional Valsalva maneuver and the modified Valsalva blowing device, raising the positive detection rate from 49.1% with the traditional maneuver to 59% with the modified device, suggesting improved sensitivity for detecting RLS with adequate execution of Valsalva. Therefore, this device may be used as an alternative method in cTCD examination, particularly in individuals unable to perform a standard Valsalva maneuver effectively [121]. In addition, recent advancements in robot‐assisted TCD have also shown higher sensitivity in detecting RLS, with a reported sensitivity of 92% and specificity of 87.5% [122].

### 6.2 TTE/cTTE

TTE has been widely used to evaluate cardiac structure and global function. Although TTE is an available technique for PFO detection, its sensitivity is low. Moreover, direct visualization of a thrombus within a PFO by TTE is extremely rare. Saleh et al. and Senapati et al. each described a very rare case of a large thrombus straddling the PFO and extending from the right atrium into the LA, as demonstrated by echocardiography, one in a 78‐year‐old man and the other in a man in his 60s [123, 124]. However, such straddling thrombi in the PFO are exceptional findings and are seldom detected with traditional TTE.

When agitated saline contrast is used, cTTE serves as a noninvasive and well‐tolerated screening tool, providing direct imaging of the shunt's anatomical location by revealing microbubbles traversing the fossa ovalis across the interatrial septum. Such visualization facilitates accurate localization of the shunt and effectively eliminates alternative explanations for bubble transit, such as early recirculation from the pulmonary veins [120]. cTTE has relatively high specificity but limited sensitivity for diagnosing RLS compared with TEE. A meta‐analysis evaluating the diagnostic accuracy of cTTE for RLS detection reported a sensitivity of 80% and a specificity of 94% [125].

Recently, several studies have explored methods to improve cTTE performance. Li et al. proposed using 50% glucose instead of agitated saline as a contrast agent during cTTE examination. Compared with saline, 50% glucose produces microbubbles with a longer peak time, longer effective duration, and longer overall duration, allowing physicians to visualize RLS across the PFO more easily [126]. However, no difference in sensitivity for detecting PFO was observed between cTTE using 50% glucose and cTTE using saline [126]. In addition, the site of contrast injection may affect cTTE performance. Studies have indicated that femoral vein injection can increase sensitivity by approximately fourfold compared with traditional antecubital vein injections [127]. Nevertheless, current guidelines do not emphasize the injection site, and further RCTs are needed to validate these findings.

### 6.3 TEE/cTEE

TEE/cTEE has been regarded as the gold standard for diagnosing PFO [13]. It provides superior visualization of the interatrial septum and detailed anatomy of PFO, which are not readily visible on TTE. However, this procedure is invasive, and the patients may experience discomfort and have difficulty performing the Valsalva maneuver during the examination. Therefore, in clinical practice, false‐positive or false‐negative results may occur due to factors such as suboptimal patient cooperation, anatomical variations, nonstandard execution of the Valsalva maneuver, or operator inexperience. These inaccuracies can lead to unnecessary invasive procedures or delayed treatment due to missed diagnosis. Phinicarides et al. retrospectively analyzed 346 patients diagnosed with PFO by TEE and found that 20 patients were ultimately identified as false positives by subsequent cardiac catheterization. Further analysis demonstrated that the use of the mid‐esophageal bicaval view was an independent predictor of diagnostic accuracy [128]. The authors suggested that combining multiple views, such as the mid‐esophageal aortic valve short‐axis view, bicaval view, and contrast bubble test, may enhance diagnostic reliability [128]. In addition, evidence indicates that combining TEE with cTTE may be more effective in identifying PFO patients at higher risk of CS [5].

On the other hand, TEE examination may carry a false‐negative rate, thereby compromising diagnostic accuracy and potentially leading to delayed diagnosis and recurrent stroke. Guan et al. performed transcatheter exploration for PFO in 30 patients with CS who had a high clinical suspicion of PFO but negative TEE results. Of these, transcatheter exploration confirmed the presence of PFO in 28 patients (93.3%), who subsequently underwent transcatheter closure, with no recurrent stroke or TIA, residual shunting, or device‐associated complications observed during 1‐year of follow‐up [129]. Therefore, in cases with strong clinical suspicion that a PFO is the underlying cause of CS despite negative TEE results, transcatheter exploration can facilitate a definitive diagnosis and help prevent missed diagnoses. However, this study is based on a limited sample size, and further large‐scale studies are required to validate its broader clinical applicability.

### 6.4 3D TEE

Given the limitations of single diagnostic techniques in accurately identifying RLS and PFO, advanced imaging approaches have been explored. Among these, 3D TEE provides an extensive set of high‐resolution ultrasound arrays and high imaging quality over traditional ultrasound imaging equipment [130]. It has been reported that 3D TEE can optimize visualization of the PFO, particularly in identifying high‐risk PFO morphological features [33]. Recently, Gwak et al. designed a practical 3D contrast‐TEE protocol using agitated saline, which significantly increased the detection rate of PFO in patients with ischemic stroke. This approach provided added value over traditional 2D imaging by improving diagnostic accuracy [131]. In addition, 3D TEE has important value in assessing PFO tunnel width and length to guide the selection of an appropriate closure device [132]. Beyond this, multimodel strategies have also been investigated. Tang et al. combined cTEE with real‐time 3D volume imaging to evaluate the characteristics of RLS in patients with CS and migraine. Compared with conventional TEE alone, this multimodel strategy significantly increased the detection rate of RLS and enabled the determination of their origin, including intracardiac shunts such as PFO and extracardiac sources such as pulmonary arteriovenous fistula, suggesting its potential in accurate identification of the types and characteristics of RLS, thereby providing additional value for clinical decision‐making [133].

### 6.5 Cardiac MRI and Computed Tomographic Angiography

Although cardiac MRI has been used to detect PFO in several studies, it remains inferior to TEE in evaluating RLS [13]. Meinel et al. conducted a meta‐analysis evaluating the diagnostic yield of cardiac MRI for identifying structural sources of embolism in patients with ischemic stroke. Compared with TEE, the detection rate of PFO was markedly lower with cardiac MRI (53.7 vs. 29.3%) [134]. Despite this limitation, cardiac MRI can provide comprehensive visualization of thoracic structures beyond the heart itself [120]. Therefore, cardiac MRI may only be used on an individualized basis, providing supplementary information on anatomical structures or tissue characteristics in specific cases [120].

Cardiac computed tomographic angiography (CTA) has also been used to detect PFO in several studies. In a study by Xiong et al., cardiac CTA demonstrated a sensitivity of 85.3%, a specificity of 71.4%, and a positive predictive value of 96.7% for PFO detection, suggesting that it may serve as an alternative imaging modality [135]. In another study by Sun et al. involving 108 patients with CS and positive findings on cTCD examination, cardiac CTA demonstrated a sensitivity of 70% and a specificity of 100% for PFO detection compared with TEE, with a positive predictive value of 100% [136]. Interestingly, the diagnostic performance of cardiac CTA for PFO detection was better in patients with moderate‐to‐large RLS than in those with small RLS [136]. Taken together, these findings suggest that while cardiac MRI and CTA are not first‐line tools for PFO detection, they may serve as a complement to conventional diagnostic approaches in selected patients with suspected PFO.

### 6.6 Intracardiac Echocardiography

Intracardiac echocardiography (ICE) enables direct visualization of PFO anatomy during cardiac catheterization and guides the selection of device size and PFO closure, without the need for general anesthesia or the involvement of an ultrasound physician [137, 138, 139]. Moreover, ICE‐guided PFO closure is associated with relatively low fluoroscopy exposure, high procedural success rates, minimal complications, and improved patient comfort, suggesting the efficiency and safety of ICE, particularly in older adults, pediatric populations, and frail populations [139]. Shatla and colleagues compared clinical outcomes, including in‐hospital adverse events, length of stay, cost, and 30‐day nonelective readmissions in patients who underwent ASD or PFO closure using TEE or ICE. Their findings showed that the ICE‐guided group had lower hospitalization costs without an increase in in‐hospital adverse events compared with those in the TEE group [140]. However, the use of ICE has several limitations. It typically requires an additional vascular access, and the operator must overcome a learning curve to achieve proficiency. Moreover, ICE‐associated procedural risks should not be overlooked, including cardiac perforation and vascular complications [130]. Following the development of 3D ICE, this technique holds promise for further improving anatomical assessment and procedural guidance in PFO closure.

**FIGURE 3:**
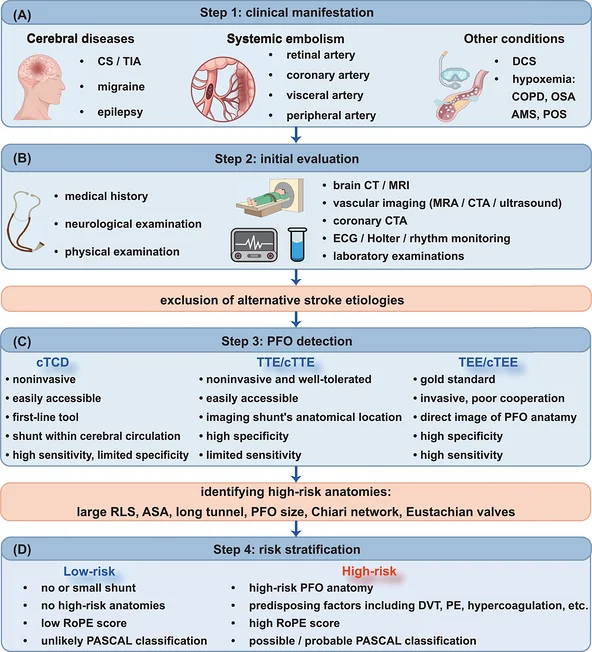
The diagnostic workflow and classification of PFO. The diagnostic workflow of PFO can be summarized into the following steps. (A) Patients presenting with cerebral diseases, such as CS/TIA, migraine, or epilepsy, systemic embolism, or other PFO‐associated conditions, such as DCS and hypoxemic diseases, undergo further evaluation. (B) The initial evaluation aims to identify potential stroke etiologies through comprehensive clinical evaluation, including medical history, careful neurological and physical examination, as well as auxiliary examinations such as brain CT or MRI, vascular imaging, coronary CTA, ECG, Holter or rhythm monitoring, and laboratory tests. (C) After exclusion of alternative stroke etiologies, PFO detection is performed using cTCD, TTE/cTTE, and TEE/cTEE, each providing complementary information on RLS level and PFO anatomy. (D) By combining the characteristics from the above steps, PFO can be divided into low‐risk (no or small shunt, no high‐risk anatomies, low RoPE score, and unlikely PASCAL classification) or high‐risk (high‐risk anatomies, presence of predisposing factors, high RoPE score, and possible/probable PASCAL classification). High‐risk PFOs may have a higher likelihood of causing symptoms. Abbreviations: AMS, acute mountain sickness; ASA, atrial septal aneurysm; COPD, chronic obstructive pulmonary disease; CS, cryptogenic stroke; CT, computed tomography; CTA, computed tomographic angiography; cTCD, contrast transcranial Doppler ultrasound; DCS, decompression sickness; DVT, deep vein thrombosis; ECG, electrocardiography; MRA, magnetic resonance angiography; MRI, magnetic resonance imaging; OSA, obstructive sleep apnea; PASCAL, PFO‐Associated Stroke Causal Likelihood; PE, pulmonary embolism; POS, platypnea–orthodeoxia syndrome; RLS, right‐to‐left shunt; RoPE, Risk of Paradoxical Embolism; TEE/cTEE, transesophageal echocardiography/contrast transesophageal echocardiography; TIA, transient ischemic attack; TTE/cTTE, transthoracic echocardiography/contrast transthoracic echocardiography.

Overall, despite variability in diagnostic strategies across different hospitals and the absence of an established optimal strategy, a widely recognized approach in clinical practice is to perform initial evaluation with cTCD, while reserving TEE for cases when further assessment is necessary [141, 142]. Developing more complementary diagnostic tools alongside TTE and TEE is expected to contribute to precise PFO detection and risk stratification. Here, we summarized the diagnostic workflow and classification of PFO in Figure 3.

## 7 The Management of PFO

**FIGURE 4:**
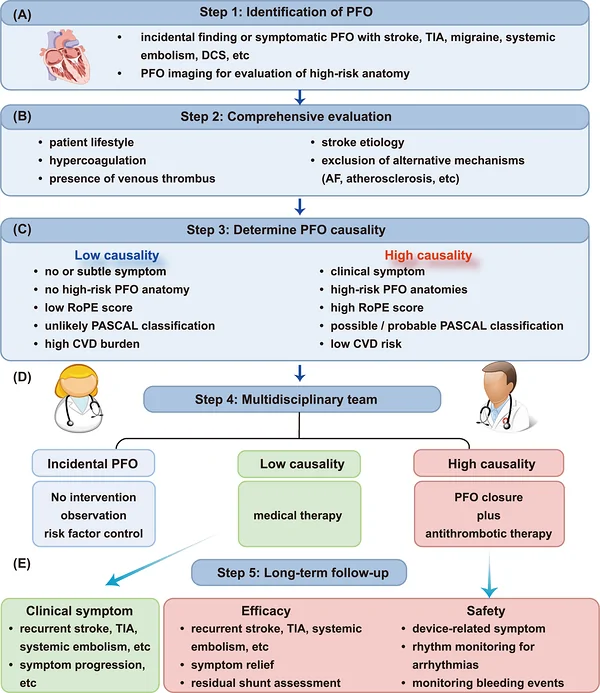
The clinical management decision‐making process for PFO. The clinical management of PFO requires a systematic and individualized process. (A) When a patient is suspected of having a PFO, clinicians should determine whether the PFO is an incidental finding or is associated with symptoms, followed by assessment of PFO morphology to identify high‐risk anatomies. (B) Then, a comprehensive evaluation should be conducted to identify predisposing factors, possible stroke etiologies, and alternative mechanisms leading to the clinical presentations. (C) After a comprehensive evaluation, the causality of PFO should be determined using risk stratification tools such as the RoPE score and PASCAL classification. The presence of clinical symptoms, high‐risk PFO anatomies, high RoPE scores, possible or probable PASCAL classification, and low CVD risk collectively indicate high causality. (D and E) Next, individualized treatment strategies should be developed by a multidisciplinary team including cardiologists, neurologists, or other symptom‐related specialists. For those with incidental PFO, intervention is generally unnecessary. For those with low‐causative PFO, medical therapy and long‐term follow‐up for recurrent events or symptom progression are recommended. For those with high‐causative PFO, transcatheter PFO closure with antithrombotic therapy should be considered. Following PFO closure, long‐term follow‐up for efficacy includes evaluation of recurrent cerebrovascular events, symptom improvement (such as migraine relief), and assessment of residual shunt. Follow‐up for safety focuses on bleeding events, arrhythmias, and device‐related symptoms such as palpitation. Abbreviations: AF, atrial fibrillation; CVD, cardiovascular disease; PASCAL, PFO‐Associated Stroke Causal Likelihood; RoPE, Risk of Paradoxical Embolism; RLS, right‐to‐left shunt; TIA, transient ischemic attack.

The optimal management of PFO remains a debated clinical challenge. Meier proposed that PFO closure acts as a form of “mechanical vaccination” and should be used for primary prevention [24]. However, this view is relatively radical and has not gained wide acceptance. In contrast, using PFO closure as a strategy for secondary prevention following PFO‐associated stroke appears more rational and is more broadly supported [21, 95]. In this review, we summarize the recent evidence and advances in the management of PFO and provide a decision‐making process for PFO (Figure 4).

### 7.1 Transcatheter Closure of PFO

#### 7.1.1 PFO Closure in Patients With CS

##### 7.1.1.1 Assessing the Relationship Between PFO and Stroke

A significant challenge is that a PFO‐associated paradoxical embolism is often a presumptive diagnosis. Not all strokes occurring in patients with PFO are attributable to the PFO itself. Given that approximately one in four adults has a PFO, in a large proportion of the population, the coexistence of PFO and stroke may be coincidental [137]. Even in the cases of CS, an identified PFO may be an incidental observation rather than the underlying pathogenic mechanism [25]. Thus, a persistent clinical issue remains how we can determine whether a detected PFO is pathogenic in a given patient and, consequently, whether closure is warranted. Identifying pathogenetic PFO is essential for selecting appropriate patients with CS for closure, maximizing clinical benefit to prevent recurrent stroke, and avoiding potentially harmful or costly overtreatment.

Currently, the most widely used tools for assessing the potential causal relationship between PFO and stroke are the Risk of Paradoxical Embolism (RoPE) score and PFO‐Associated Stroke Causal Likelihood (PASCAL) classification system. The RoPE score estimates the probability that a PFO is responsible for a CS, with higher scores indicating a greater probability of causality [143]. However, the RoPE score has several limitations, including the lack of anatomical features of the PFO and the inability to predict the risk of recurrent strokes [141]. A recent study reported that the RoPE scores can also predict high‐risk anatomical and physiological features of PFO, suggesting potential utility beyond its original scope [144]. To address these limitations of the RoPE score, the PASCAL system was developed by combining the RoPE score items with anatomical features of PFO. This classifies patients into three categories, including unlikely, possible, or probable, regarding whether the PFO is the cause of CS [49, 145]. The PASCAL classification offers a reasonable and pragmatic approach to guide clinical decision‐making. Evidence from a meta‐analysis showed that the PASCAL classification algorithm can distinguish candidates who are likely to benefit from PFO closure from those who may not among young and middle‐aged patients with PFO and otherwise CS [146]. Specially, PFO closure was associated with a relative risk reduction for recurrent ischemic stroke of 90% in the probable group and 62% in the possible group, whereas no significant advantage over medical therapy alone in the unlikely group [141]. The combination use of these tools facilitates evidence‐based decisions of PFO closure. Nevertheless, the PASCAL system also has limitations, including its unsuitability for patients older than 60 years or pediatric individuals, and the lack of prospective validation [49].

Given the constraints of the RoPE and PASCAL systems, developing a new risk stratification system to predict high‐risk PFO patients with a greater risk of stroke is critical. Rigatelli et al. developed the CRISP‐PFO score, identifying the following independent predictors of CS, including permanent RLS, presence of ischemic brain lesions, left atrial diameter ≥43 mm, and ASA. Based on these parameters, patients were classified into low‐, intermediate‐, and high‐risk categories, with progressively increasing event rates across groups. Therefore, this scoring system can help identify PFO patients at high risk of CS [147], but large‐scale validation studies are required before it can be widely used in clinical practice.

##### 7.1.1.2 Evidence for PFO Closure in CS

Epidemiologic data indicate that 12% of patients who experience an acute ischemic stroke will have a recurrence within 5 years [148]. Stroke recurrence remains a major concern in the management of ischemic stroke, as recurrent events tend to result in greater disability and higher mortality than initial strokes, adversely affecting both quality of life and long‐term outcomes.

Three high‐quality RCTs have demonstrated that transcatheter PFO closure is superior to medical therapy alone in reducing stroke recurrence in patients with CS [10, 11, 12] (Table 1). Subsequent observational studies, retrospective analyses, and meta‐analyses have consistently confirmed that PFO closure markedly reduced the risk of recurrent ischemic stroke or TIA and all‐cause mortality compared with antiplatelet or anticoagulant therapy [149, 150, 151, 152]. Based on the results, current guidelines have recommended transcatheter PFO closure as an established therapy for secondary stroke prevention in patients aged 18–60 years with prior PFO‐related stroke [21, 95]. However, there is no supporting evidence for PFO closure in primary prevention, and it is therefore not recommended for that indication.

**TABLE 1:** Completed and ongoing RCTs regarding PFO closure in patients with CS.

		Start year				Experimental group	Control group		Primary end point	
Number	Trial		Enrollment	Age	Follow‐up		Antiplatelet	Anticoagulation		Results
NCT00166257	PC‐Trial [159] *n* = 414	2000	PFO + CS, TIA, or peripheral thromboembolism	18–60y	4.0 years	Closure AMPLATZER PFO occluder	Aspirin 100–325 mg/d or clopidogrel 75–150 mg/d	Anticoagulation	A composite of death, nonfatal stroke, TIA, or peripheral embolism	Closure did not significantly reduce recurrent embolic events or death.
NCT00562289	CLOSE [11] *n* = 663	2007	PFO with ASA and large RLS + CS	16–60y	5.3 ± 2.0 years	Closure, DAPT (aspirin + clopidogrel) for 3 months, SAPT throughout the remainder of the trial	Aspirin, clopidogrel, or aspirin + dipyridamole	Vitamin K antagonists, dabigatran, apixaban	Occurrence of stroke	Closure reduces stroke recurrence rate.
NCT00465270	RESPECT [12, 160] *n* = 980	2003	PFO + CS	18–60y	2.6 ± 2.0 years (2013) 5.9 years (2017)	Closure AMPLATZER PFO occluder	Aspirin, clopidogrel, or aspirin + dipyridamole, aspirin + clopidogrel	Warfarin	Composite of recurrent nonfatal/fatal ischemic stroke or early death	Closure reduces recurrent ischemic strokes more than medical therapy alone.
NCT01550588	DEFENSE‐PFO [161] *n* = 120	2012	CS + high‐risk PFO	18–80y	2.8 years	Closure AMPLATZER PFO occluder	Aspirin, aspirin + clopidogrel, cilostazol	Warfarin	Composite of stroke, vascular death, or TIMI‐major bleeding	Closure reduced primary endpoint as well as stroke recurrence.
NCT00738894	REDUCE [10] *n* = 664	2008	CS + PFO	18–60y	3.2 years	PFO closure with septal occluder device	Aspirin, clopidogrel, or aspirin + dipyridamole	NA	Freedom from stroke, incidence of new brain infarction on imaging	Closure reduces recurrent ischemic strokes more than medical therapy alone.
NCT05387954	CLOSE‐2	2023	PFO with ASA and large RLS + CS	60–80y	At least 4 years	PFO closure + DAPT (aspirin + clopidogrel) for 3 months, +SAPT (aspirin or clopidogrel)	DAPT (aspirin + clopidogrel) for 3 months, +SAPT (aspirin or clopidogrel)	Apixaban, dabigatran, rivaroxaban	Time to recurrent stroke (ischemic or hemorrhagic fatal or nonfatal)	Ongoing
NCT05907694	STOP	2024	PFO with RLS + CS	>60y	At least 12 months to 10 years	PFO closure + SAPT + risk factor control	SAPT + risk factor control	NA	Rate of ischemic events	Ongoing
NCT07300358	BIOCLOSE‐PFO	2025	PFO with a moderate‐to‐large RLS + CS/TIA	18–65y	At least 12 months	Biodegradable PFO occluder system vs. Cardi‐o‐fix PFO occluder (control)	NA	NA	Success rate of effective occlusion	Ongoing
NCT04475510	HALTI Trial	2020	CS/TIA + PFO closure	18–60y	12–24 months	Antiplatelet treatment discontinuation at 12 months post‐PFO closure	NA	NA	New stroke events and MRI lesions within 12 months after discontinuation	Ongoing

Abbreviations: ASA: atrial septal aneurysm; CS: cryptogenic stroke; DAPT: dual antiplatelet therapy; RCT: randomized controlled trials; RLS: right‐to‐left shunt; SAPT: single antiplatelet therapy; TIA: transient ischemic attack.

Although the efficacy and safety of PFO closure in patients with CS have been demonstrated in a series of studies [10, 11, 12, 152], these clinical trials had a mean follow‐up duration of less than 6 years, highlighting the need for more extensive long‐term outcome real‐world data on PFO closure. In 2025, the PROLONG registry reported long‐term outcomes of PFO closure using a large‐scale multicenter national cohort by retrospective analyses of 1245 patients with cryptogenic embolism (including CS or TIA, systemic embolism, and silent ischemic lesions on MRI) who underwent PFO closure, with a mean follow‐up duration of 14.5 ± 2.4 years. Only 34 patients (2.7%) experienced recurrent stroke, TIA, or systemic embolism, and serious procedure‐associated complications were rare both during hospitalization and at follow‐up, suggesting the long‐term efficacy and safety of PFO closure. The study identified predictors of recurrence during follow‐up, including a lower RoPE score (≤7), nonprobable PASCAL classification, and new‐onset AF [153]. Similarly, Angerbjörn et al. enrolled 827 patients with CS or TIA who underwent PFO closure and were followed for a median of 8 years. Patients who received PFO closure had lower all‐cause mortality compared with the general population [154]. In a retrospective study with a median follow‐up of 10 years enrolling 330 patients with stroke or TIA who underwent PFO closure by Goessinger et al., 3.6% experienced recurrent stroke, TIA, or stroke‐related death, corresponding to a recurrence rate of 0.38 per 100 patient‐years [26]. Further analysis showed that the RoPE score, PASCAL classification, and a history of prior neurological events were independent predictors of subsequent recurrent neurologic events, but age, sex, cardiovascular risk factors, and new‐onset AF showed no effects [26]. Moreover, a retrospective study including 130 patients with CS, TIA, or peripheral embolism who underwent PFO closure was performed with up to 20 years of follow‐up to evaluate clinical outcomes. After follow‐up, recurrent ischemic events were observed in only one patient with stroke and six patients with TIA, indicating an extremely low rate of recurrence, particularly recurrent stroke (<1%). Although more than one‐fifth of patients discontinued antithrombotic therapy, no ischemic events occurred among them [155]. A first and comprehensive review of the previous studies aimed to evaluate long‐term adverse outcomes in adults who underwent PFO closure by Asghar et al. further demonstrated a low recurrent stroke rate of 0.34 per 100 person‐years over follow‐up periods ranging from 4 to 12.3 years, consistent with randomized trial data [152]. These findings underscore the very long‐term efficacy and safety of the procedure.

Despite these favorable outcomes, the risk of recurrent ischemic stroke after PFO closure remains higher than that in the general population. Bonnesen et al. evaluated stroke recurrence after PFO closure using nationwide population‐based Danish registries and found a 2.1% higher absolute risk of ischemic stroke within 4 years among patients who underwent PFO closure compared with the controls [156]. The residual risk may be related to anatomical factors. For example, Sun et al. enrolled 247 PFO patients with CS who underwent PFO closure and found that patients with complex PFO had a higher adverse event incidence, with complex PFO independently associated with poorer outcomes [157]. In addition, the traditional risk factors still influence long‐term outcomes after PFO closure [154]. In a study by Sørensen et al., among 310 patients who underwent PFO closure, 2.6% of the patients experienced recurrent stroke or TIA during a median follow‐up of 2.6 years. They found that recurrence was more frequent in patients with hypertension or in patients with thrombophilia or hematologic disorders predisposing to hypercoagulability [151]. This suggests that stroke/TIA recurrence may be associated with preexisting risk factors (such as hypertension) and a hypercoagulable state. Therefore, patients who are projected to receive closure should continue to pay attention to the management of these traditional risk factors.

The timing of PFO closure after CS remains a topic of debate and lacks direct recommendations. A statement from an expert consensus suggested that PFO closure should be considered within 6 months of the index stroke [21]. However, a multicenter retrospective study by Jerónimo et al. involving 496 patients with cryptogenic CVEs or systemic embolism found no significant differences in recurrence (3.9 vs. 2.6%) or mortality (1.4 vs. 1.0%) between early (<9 months) and delayed (≥9 months) closure during a median follow‐up of 2 years. Surprisingly, even very delayed closure (≥24 months) also showed no difference in recurrence rate compared with closure within 24 months (4.2 vs. 3.4%) [158]. These findings suggest that patients with remote CVE or systemic embolism may still benefit from PFO closure, and that treatment decisions should be individualized based on clinical symptoms and cerebral infarct characteristics.

#### 7.1.2 PFO Closure in Patients With Migraine

The evidence regarding the efficacy and potential benefit of PFO closure for migraine remains inconsistent and considerably debated (Table 2). Three prior RCTs, including MIST, PRIMA, and PREMIUM, failed to demonstrate a superiority of PFO closure over medical therapy for migraine treatment [162, 163, 164]. However, although the PREMIUM trial failed to meet its primary efficacy endpoint, subgroup analysis showed that patients who underwent closure experienced a significant reduction in headache days compared with those receiving medical therapy alone [164].

**TABLE 2:** Completed and ongoing RCTs regarding PFO closure in patients with migraine.

		Start year				Experimental group	Control group		Primary end point	
Number	Trial		Enrollment	Age	Follow‐up		Antiplatelet	Anticoagulation		Results
ISRCTN 45687883	MIST [162] *n* = 147	2004	Migraine with aura + moderate or large RLS with PFO	18–60y	180 days	Closure + aspirin and clopidogrel in the 24 h before the procedure (300 mg each) and for 90 days after the procedure (75 mg)	Sham procedure + aspirin and clopidogrel in the 24 h before the procedure (300 mg each) and for 90 days after the procedure (75 mg)	NA	Cessation of migraine headache 91–180 days after the procedure	No significant difference between the two groups
NCT00505570	PRIMA [163] *n* = 107	2006	Refractory migraine with aura + PFO	18–65y	12 months	PFO occluder + aspirin 75–100 mg for 6 months and clopidogrel 75 mg for 3 months	Aspirin 75–100 mg for 6 months and clopidogrel 75 mg for 3 months	NA	Reduction in monthly migraine days during Months 9–12 after randomization	Closure did not reduce overall monthly migraine days.
NCT00355056	PREMIUM [164] *n* = 230	2006	Migraine refractory to drugs + PFO	18–65y	12 months	Pretreated with aspirin 325 mg and clopidogrel 600 mg + PFO closure	Pretreated with aspirin 325 mg and clopidogrel 600 mg + sham procedure	NA	Percentage of subjects with a 50% reduction from baseline in the monthly number of migraine attacks	Closure did not meet the primary endpoint of reduction.
NCT03521193	LEARNER [171] *n* = 78	2018	Migraine with aura + PFO closure + aspirin	18–70y	12 months	Closure + DAPT 2 months + aspirin	Healthy subjects on aspirin	NA	Change in migraine characteristics at 6‐months and 12‐months	MHA‐PFO patients display an increased prothrombotic phenotype, reversed by closure.
NCT02127294	EASTFORM [172] *n* = 241	2013	Migraine + PFO with a moderate to large RLS	18–65y	1 year	Closure + aspirin for 6 months	No closure	NA	RLS evaluation via cTCD and HIT‐6 scores within 1 year postprocedure	Closure reduced migraine
NCT05546320	COMPETE [173]	2022	Migraine + PFO with RLS	18–64y	12 weeks	Metoprolol (positive control)	Aspirin, clopidogrel	Rivaroxaban	A ≥50% reduction in the mean number of monthly migraine days or migraine attacks	Aspirin, clopidogrel, rivaroxaban were all noninferior to metoprolol in the primary endpoint.
NCT05561660	COMPETE‐2	2022	Migraine + PFO	18–65y	12 months	Closure	Aspirin 200 mg for 6 months	NA	50% migraine reduction and safety assessment	Ongoing
NCT04946734	SPRING	2021	Migraine + PFO with RLS	16–60y	12 months	Closure + DAPT (aspirin + clopidogrel) for 1 month + aspirin for 6 months + Triptans prn	DAPT (aspirin + clopidogrel) for 1 month + aspirin for 6 months + Triptans prn	NA	Complete cessation of migraine; serious adverse event	Ongoing
NCT06203873	BioMetal	2024	Severe migraine + PFO with RLS	18–65y	12 months	Biodegradable occluder	Metal occluder	NA	Average reduction in monthly migraine days	Ongoing

Abbreviations: cTCD: contrast transcranial Doppler ultrasound; DAPT: dual antiplatelet therapy; RCT: randomized controlled trials; RLS: right‐to‐left shunt; MHA: migraine with aura.

Encouraged by these findings, a growing number of retrospective, nonrandomized observational studies and several meta‐analyses have focused on the role of PFO closure in treating migraine and support that the procedure may reduce migraine frequency and severity in selected patients [66, 165, 166]. In a study by Sun et al., among 172 migraine patients with PFO, those who underwent PFO closure showed a significant reduction in migraine severity and anxiety/depression at the 1‐ and 3‐month postoperative follow‐ups. Further analysis revealed that migraine with aura, intrinsic RLS, and higher baseline HIT‐6 scores were consistent predictors of symptomatic improvement [66]. These findings confirmed the short‐term effectiveness of PFO closure in alleviating migraine symptoms. Subsequently, a recent study from Japan evaluated the efficiency of PFO closure in patients with drug‐resistant migraine. Among 27 patients who experienced more than two migraine attacks per month despite medication, 22 (81%) patients achieved complete resolution or improvement at 12 months. Notably, nearly half of the patients (48%, 10 out of 21) with aura experienced complete resolution, whereas those without aura showed a much lower response rate, with only one case of complete remission [167]. Thus, PFO closure may be more suitable for patients with migraine with aura.

It should be noted that the aforementioned studies are limited by relatively small sample sizes. To address this, larger pooled analyses and meta‐analyses have been conducted. Based on an analysis of 1165 participants from three RCTs, one pooled study, and eight retrospective case series, Zhang et al. reported that PFO closure markedly reduced headache frequency, monthly migraine attacks, and monthly migraine days compared with control interventions. Moreover, patients with migraine with aura were more likely to achieve headache resolution after PFO closure [165]. Similarly, another meta‐analysis including five RCTs and six observational studies showed that PFO closure was associated with a significant reduction in migraine attacks and migraine days per month compared with medical therapy alone. However, no significant differences were observed between groups in complete migraine resolution, HIT‐6 scores, or MIDAS scores [166]. These findings suggest that PFO closure may be a promising and alternative option for migraine treatment.

However, the efficacy of PFO closure in migraine is not consistent across all cases. White matter lesions (WMLs) are commonly observed in migraine patients, suggesting potential risks of cognitive impairment and stroke [62]. In a study by Yan et al., 78 migraine patients with massive RLS who underwent PFO closure were divided into two groups according to the presence or absence of WMLs. Both groups showed a reduction in postoperative migraine symptoms. However, compared with patients without WMLs, those with WMLs exhibited a higher frequency and longer duration of headache episodes, along with higher HIT‐6 and VAS scores, indicating a poorer clinical efficacy. The presence of WMLs has been considered an independent predictor of reduced efficacy following PFO closure [168]. Therefore, although PFO closure may be beneficial for migraine patients with RLS, its efficacy may be attenuated in those with coexisting WMLs. In such cases, clinical focus should be placed on the management of WMLs themselves, rather than merely performing closure blindly.

Not all migraine patients derive benefit from PFO closure, highlighting the importance of appropriate patient selection to optimize clinical outcomes. Recently, Wang et al. developed a nomogram model to predict migraine remission after PFO closure. Their findings revealed that high MIDAS scores before closure, the presence of mitigating factors, lower monthly attack frequency, TCD findings, and elevated plateletcrit were independent predictors [169]. In another Chinese retrospective study of 139 migraine patients who underwent PFO closure, the independent risk factors for postoperative headache nonremission were identified as smoking history, AF, absolute lymphocyte count, platelet‐to‐lymphocyte ratio, and interventricular septal thickness [170].

The pathogenesis of migraine is multifactorial and intricate, and the PFO‐mediated mechanism represents only one of the potential pathways. Therefore, PFO closure is unlikely to benefit all patients with migraine, and a personalized therapeutic strategy remains critical. To date, no RCT has met its primary endpoint in supporting PFO closure as an established treatment for migraine. Accordingly, whether PFO closure can effectively reduce the burden of migraine remains controversial. Current guidelines do not recommend routine screening for PFO in patients with migraine, nor do they support PFO closure as a standard therapeutic approach [95]. Nevertheless, considering the emerging evidence from retrospective studies and meta‐analyses of RCTs has suggested that PFO closure may improve migraine symptoms, including reductions in migraine days, attack frequency, and overall severity, some investigators propose that PFO closure may be beneficial in selected patients with migraine [34]. However, this notion remains controversial, and a substantial proportion of experts hold opposing opinions. Future well‐designed RCTs are required to investigate the causal link between PFO and migraine, and to accurately identify patients who are most likely to benefit from PFO closure. In particular, it remains necessary to distinguish between migraine patients with pathogenic PFO and those in whom PFO is incidental.

#### 7.1.3 PFO Closure in Patients With Systemic Embolism

Besides stroke and migraine, PFO closure has been proposed for other indications, including systemic embolism in noncerebral vascular territories. Given the shared mechanism underlying PFO‐related stroke and systemic embolic events, PFO closure may be reasonable in patients who experience PFO‐related systemic embolism after other possible embolic etiologies have been excluded [95]. A statement from the SCAI guideline suggests that PFO closure should be considered in patients with systemic embolism and no identifiable embolic etiology [95]. However, since current evidence is mainly derived from case reports or small observational studies, the benefit of PFO closure in this population remains to be validated by larger, well‐designed studies.

#### 7.1.4 Antithrombotic Strategy After PFO Closure

Guidelines recommend that patients receive antiplatelet therapy following PFO closure [21, 95]. After device implantation, complete endothelialization is typically achieved within 6 months, although in some cases it may take up to 5 years [21]. Antiplatelet therapy is critical in promoting endothelial coverage of the device and protecting against ischemic events. Therefore, an expert consensus statement suggests that patients with CS undergoing PFO closure should receive dual antiplatelet therapy (DAPT) for 1–6 months, followed by long‐term single antiplatelet therapy for at least 5 years [21]. However, evidence regarding the optimal duration of postclosure antithrombotic therapy remains limited, and longer‐term strategies are still not well defined. In a study by Srivastava et al., no device thrombosis or embolization was observed during the 1‐year follow‐up, with a mean DAPT duration of 2.91 ± 2.6 months, suggesting that a DAPT duration ≤3 months may be sufficient in patients after PFO closure [174]. In contrast, a meta‐analysis by Lim et al. revealed that patients with thrombophilia have an increased risk of recurrent stroke or TIA after PFO closure compared with those without thrombophilia [175]. This raises a concern about whether this high‐risk population requires extended DAPT, and whether antiplatelet or anticoagulation constitutes the appropriate antithrombotic strategy.

Importantly, long‐term antithrombotic therapy may pose an increased risk of bleeding. In a 20‐year follow‐up study after PFO closure, bleeding events occurred in 17 (13%) patients, more than half of which were major bleeding, and all were observed in individuals receiving long‐term antithrombotic therapy. Notably, women experienced more bleeding events than men (19.4 vs. 6.3%) [155]. Therefore, it may be important to individualize the duration of antithrombotic therapy following PFO closure based on factors such as age, sex, and cardiovascular risk profile, particularly in patients with high bleeding risk and low risk of ischemic recurrence [155].

To date, no studies have directly compared outcomes of different durations of antiplatelet therapy or the use of different antiplatelet drugs after PFO closure. Consequently, evidence regarding the optimal postprocedural antithrombotic strategy remains lacking. Future well‐designed studies are warranted to provide evidence‐based recommendations on the optimal duration and choice of postprocedure antiplatelet therapy to balance the prevention of recurrent ischemic events with the risk of bleeding complications.

### 7.2 New Advancements in Occluders

#### 7.2.1 The Disadvantage of Metal Occluders

Traditional metal occluders for PFO closure, such as the Amplatzer PFO Occluder, are nickel‐containing devices. They are easy to deploy, have favorable safety, and have been widely adopted in clinical practice. However, concerns persist regarding the properties of nitinol, particularly nickel allergy, which is one of the most common contact allergies worldwide [176]. As a well‐recognized allergen, nickel can induce Type IV hypersensitivity reactions in more than 20% of the population [177, 178]. Notably, there is a clear sex disparity, with a higher prevalence of nickel allergy among women [178]. Evidence suggests that nickel allergy may be associated with a range of systemic symptoms following PFO closure, such as chest pain, palpitations, migraines, dyspnea, and cutaneous eruptions, collectively referred to as device‐related symptoms [176]. Importantly, there are currently no available validated tools for predicting such hypersensitivity reactions.

The INSPIRE trial, a double‐blind randomized study of 96 patients with CS receiving PFO closure, first explored the influence of nickel hypersensitivity. Among these patients, 28 (29.2%) were identified as having nickel hypersensitivity. Compared with patients without hypersensitivity, nickel‐hypersensitive patients had an increased rate of device‐related syndrome (71.4 vs. 20.6%), particularly new‐onset or worsening migraines and palpitations (Table 3) [177]. Interestingly, nickel hypersensitivity was more common in women, who also exhibited a higher frequency of device‐related symptoms than men [178]. In addition, the nonbiodegradable nature of traditional metal occluders may limit future transseptal puncture procedures, such as catheter ablation or left atrial appendage closure. Considering the increased risk of device‐related complications in patients with nickel hypersensitivity, it is important to optimize device selection to improve outcomes in this population.

**TABLE 3:** Completed and ongoing RCTs regarding PFO closure in patients with OSA, epilepsy, and other conditions.

Number	Trial	Start year	Enrollment	Age	Years	Experimental group	Control group	Primary end point	Results
NCT01780207	Impact of closure of patent foramen ovale on apnea–hypopnea index, nocturnal hypoxemia and systemic vascular function in patients with obstructive sleep apnea [186]	2013	Patients with moderate to severe OSA	>18y	3 months	OSA with PFO: PFO closure *n* = 14	OSA without PFO: observation *n* = 26	Apnea–hypopnea index, apnea index	Closure in OSA patients improves sleep‐disordered breathing and nocturnal oxygenation.
NCT02771561	PCOSA‐1 [187] *n* = 26	2016	OSAHS + moderate to large PFO	>18y	6 months	Single‐arm: PFO closure	NA	Change in epworth sleepiness scale/apnea–hypopnea index/oxygen desaturation index/6 min walk test/sleep apnea quality of life index (SAQLI)/functional outcomes of sleep questionnaire (FOSQ)	Closure did not improve respiratory polygraphy measures of OSAS severity.
NCT04713683	INSPIRE [177] *n* = 96	2020	PFO + CS/stroke + closure	>14y	90 days	Closure + DAPT (aspirin + clopidogrel) for 3 months	NA	The incidence of device syndrome, a composite of patient‐reported symptoms (chest pain, palpitations, migraines, dyspnea, and rash).	Patients with nickel hypersensitivity are at significantly higher risk of developing device syndrome.
NCT06863350	PREDICT‐PFO	2025	Drug‐resistant epilepsy + PFO with RLS (Grade II or higher)	18–60y	48 weeks	PFO closure	Drugs	Percentage decrease in seizure frequency	Ongoing

Abbreviations: CS: cryptogenic stroke; DAPT: dual antiplatelet therapy; OSA: obstructive sleep apnea; OSAHS: obstructive sleep apnea–hypopnea syndrome; RCT: randomized controlled trials; RLS: right‐to‐left shunt; TIA: transient ischemic attack.

#### 7.2.2 Biodegradable Occluders

In recent years, the management of PFO has evolved from traditional metal occluders toward more physiologically aligned concepts, such as “intervention without implantation” or “implantation without residue.” Biodegradable occluders, as a new‐generation closure device, can promote endothelialization and the formation of a native tissue barrier, potentially preserving access for future transseptal interventions [14]. Several recent studies have directly compared the efficiency and safety of this novel occluder and traditional metal device. In a multicenter RCT conducted by Zhang et al., 190 patients aged 18–65 years were randomly assigned to undergo PFO closure with either a biodegradable device or a nitinol device. At 6 months, the postoperative success rates, defined as RLS Grade 0 or 1 on cTTE, showed no difference between the two groups (90.63 vs. 91.49%). During the entire study period, neither group experienced any death, embolism, device‐related thrombus, or erosion. These findings indicate that biodegradable occluders are noninferior to traditional nitinol devices in terms of both efficacy and safety. Notably, the biodegradable device was completely degraded and no longer detectable on echocardiography within 24 months after implantation [14].

Current use of metallic occluders has shown benefits in patients with migraine. However, concerns regarding long‐term complications and the possibility of metallic devices exacerbating symptoms have raised a key question: can biodegradable occluders provide comparable benefits while avoiding the long‐term risks associated with permanent implants? In a retrospective cohort study, Li et al. evaluated 158 patients who underwent PFO closure using either nitinol (*n* = 77) or biodegradable (*n* = 81) occluders and found that both groups experienced significant alleviation of migraine severity as reflected by reduced MIDAS scores. Both groups achieved a 100% procedural success rate with a very low incidence of complications. After a follow‐up of 12 months, residual shunting was minimal and comparable between groups, with 82.35% of patients in the nitinol group and 78.87% in the biodegradable group presenting with Grades 0 or 1 RLS [179]. These findings suggest that biodegradable occluders appear equally effective and safe for the treatment of PFO‐associated migraine. Consistently, another single‐center study by Sun et al. reported comparable efficacy in improving migraine symptoms and similar safety profiles between biodegradable and metallic occluders [180]. However, it should be noted that the available evidence is mainly from single‐center retrospective studies with relatively small sample sizes, which may bring inevitable bias. A prospective, multicenter, single‐blind RCT (BioMetal trial) is currently underway to directly compare biodegradable and metallic occluders in patients with PFO and medication‐refractory migraine with aura [181]. The results of this trial are expected to provide valuable evidence for the clinical management of this population (Table 3).

Overall, the application of biodegradable occluders appears to be safe and effective for PFO closure, with the advantage of eliminating the potential long‐term risks associated with permanent cardiac implants, thereby offering a more appropriate therapeutic strategy for specific patient populations, such as those with nickel allergy. Although echocardiography suggests complete device degradation, direct verification by pathological gold‐standard methods is lacking. Furthermore, its long‐term safety and stability still require validation in larger‐scale cohorts with extended follow‐up.

#### 7.2.3 Suture System

In recent years, the percutaneous suture system has emerged as an alternative and effective method for PFO closure in clinical practice. Several studies compared the efficiency and safety of this procedure with those of traditional device closure. In a single‐center, retrospective study involving 55 patients undergoing PFO closure, both the suture and device groups achieved a 100% procedural success rate, with no procedure‐related complications observed [182]. Notably, patients who underwent suture‐based closure experienced no occurrence of AF, compared with three cases in the device closure group [182]. Although the absence of AF events in the suture group is clinically noteworthy, the small sample size limits definitive conclusions regarding AF risk reduction by suture technique. Recently, Gaspardone et al. conducted the largest cohort study to date, including 703 individuals with stroke or TIA who underwent suture‐mediated PFO closure. During a median follow‐up of 4 years, no recurrent CVEs were observed, and only one case of transient AF was reported, highlighting the long‐term efficacy and safety of this technique [15]. However, residual shunting remained an important limitation when suturing the PFO, occurring in 12.9% of patients. Independent predictors of residual shunt included maximum width, minimum length, ASA, and Grade 3 shunting during the Valsalva maneuver [15].

It is worth noting that patient selection is critical for suture‐mediated closure. Although TEE is necessary for anatomical assessment, it is insufficient alone, and intraoperative balloon sizing is also required [183]. Cannata et al. argued that the presence of an ASA, profound fossa ovalis excursion, an excessively wide or abnormally short PFO tunnel, and a massive RLS all contribute to procedural complexity and a higher risk of incomplete closure [183]. Compared with traditional devices, the suture‐based approach restores a more physiological septal anatomy without leaving an implant in the heart [15], thereby avoiding nickel exposure and potentially reducing the need for prolonged antithrombotic therapy [183]. However, current evidence is primarily from observational studies that did not include a control group at design, and there is no RCT directly comparing suture‐mediated and device‐based closure. Therefore, whether suturing can be applied to all PFO morphologies or replace traditional metal occluders remains uncertain.

Taken together, innovative techniques such as biodegradable occluders and transcatheter suture techniques have gradually been applied in clinical practice, marking the entry of this field into a new era of precision medicine. These approaches offer several advantages over traditional metal occluders, including avoidance of permanent metal implants, suitability for patients with nickel allergy or hypersensitivity, and preserving a clear and unobstructed structure of the interatrial septum for future transseptal interventions. Nevertheless, large‐scale randomized studies are required to validate their long‐term safety, efficacy, and especially their valid difference in procedure‐related AF incidence.

#### 7.2.4 Other Techniques

Recently, Wang et al. designed a 4D‐printed intelligent reconfigurable PFO occluder based on a biomimetic design, characterized by proendothelialization, antithrombosis, and absorbability. Moreover, it integrates mechanical occlusion with localized drug delivery, providing a promising strategy for PFO management [184]. Nevertheless, this occluder remains in the early stages of development and still requires clinical validation.

In addition, Deng et al. performed cryoablation to close PFO in patients with AF who underwent pulmonary vein isolation. The PFO closure rate at 6 months was 63.6%, with no ischemic stroke events observed during 1‐year follow‐up [185]. However, this approach demonstrated a significantly lower closure rate compared with conventional device‐based closure. Furthermore, the study was limited by its single‐center design and small sample size, which may introduce bias.

### 7.3 The Management of PFO in Special Populations

#### 7.3.1 Older Population

The role of PFO in older patients with CS has attracted increasing attention. In patients aged 60 years and older, nearly one‐third of ischemic strokes are classified as cryptogenic [137]. Notably, PFO does not spontaneously close in this age group [188]. Evidence indicated that the prevalence of PFO is significantly higher in older patients with CS than in those with an identified stroke etiology (37.1 vs. 11.8%), a pattern consistent with that observed in younger populations (56.6 vs. 18.8%) [189].

Theoretically, PFO serves as a conduit for paradoxical embolism and may represent a central pathophysiology contributor to CS in the elderly [190]. Moreover, older individuals are more prone to venous thrombosis and pulmonary hypertension, factors that further heighten the risk of PFO‐related stroke [141]. Notably, older patients with both PFO and CS appear to have a relatively high rate of recurrent ischemic stroke or TIA [191], with age itself identified as an important determinant of recurrence [192].

Although high‐risk PFOs are also commonly observed in older patients with CS, their anatomical characteristics may differ from those in younger individuals [193]. Studies have shown that large PFO size and the presence of ASA remain associated with CS in the elderly [194]. Moreover, septal malalignment is more prevalent in patients aged ≥60 years than in those under 60 years old [193]. Although older patients still have a higher incidence of ASA than younger patients (<60 years) [193, 195], the strength of the association between PFO with concomitant ASA and CS is weaker in older patients than in the younger population [137].

Given the established benefit of PFO closure in younger patients, it may be a rational choice for older patients due to the potential benefit. Several observational studies and meta‐analyses suggest that appropriately selected older patients may also benefit from PFO closure. Eichelmann et al. reported that among 342 patients with CS aged over 60 years, those who underwent PFO closure (three out of 143, 2%) had a lower rate of recurrent stroke, compared with those receiving medical therapy alone (11 out of 199, 6%) after an average follow‐up of 5.5 ± 1.5 years [196]. Consistent findings were observed in subsequent studies by Chen et al., reporting reductions in recurrent cerebral ischemic events and all‐cause mortality during an average follow‐up of 2.5 years [197], as well as by Wang et al., reporting a lower incidence of recurrent stroke [198]. In addition, Chen et al. observed better functional outcomes in elderly patients who underwent PFO closure after 180 days than those with medical therapy alone [197]. Moreover, patients older than 65 years showed an even greater reduction in recurrence rate, compared with patients aged 60–65 years old [198]. In terms of safety, procedure‐related adverse events and AF were similar between elderly and younger patients [196, 197]. Notably, patients older than 70 years had a comparable incidence of procedure‐related adverse events and AF compared with those aged 60–70 years [198].

Meta‐analyses further support these observations. One analysis showed significantly lower risks of recurrent stroke (5.48 vs. 10.05%) and all‐cause mortality (1.73 vs. 7.59%) in patients aged over 60 years undergoing PFO closure compared with those receiving medical therapy alone [199]. Another meta‐analysis reported a 45% reduction in recurrent cerebral ischemia and an 85% reduction in mortality in this population [200]. Recently, a large‐scale real‐world study from the United States, including 20,999 patients aged over 60 years, confirmed that PFO closure significantly decreased recurrent ischemic stroke compared with medical therapy alone, while maintaining a clinically acceptable safety profile [201]. These findings demonstrate that the superiority of PFO closure over medical therapy alone can extend to patients over 60 years of age, suggesting that appropriately selected older adults may also derive benefit from PFO closure.

Several studies have directly compared outcomes between older and younger patients (older or younger than 60 years) undergoing PFO closure. Overall, procedural safety and efficacy appear comparable across age groups. Nachoski et al. found no significant differences in stroke or TIA recurrence, cardiac death, arrhythmias, or residual shunt between patients aged ≥60 years and those <60 years during a median follow‐up of 3.6 ± 1.2 years after PFO closure [202]. Gili et al. extended the age range, defining the elderly patients as those over 65 years, reporting no recurrent stroke or systemic embolism during follow‐up in either the younger or the elderly group. In addition, the procedural success rate was 100% in both groups, and the complication rates, including new‐onset AF, supraventricular arrhythmias, and device embolization, were comparable between the two groups [195]. Thus, akin to their younger counterparts (18–60 years), PFO closure proves to be a safe and effective strategy for preventing recurrence in older patients, and age alone should not be considered a contraindication to PFO closure.

Despite these encouraging findings, several important limitations should be acknowledged. First, available evidence is predominantly from retrospective and observational studies, with relatively small sample sizes, and no RCT has specifically evaluated PFO closure in older patients. Second, older patients often present with multiple comorbidities or risk factors, and alternative cerebral embolic sources, including aortic atheroma and AF, may exist and confound the causal relationship for PFO. Indeed, one retrospective study, enrolling 1090 patients with ischemic stroke aged over 65 years, found no difference in stroke recurrence between elderly patients with and without PFO. Instead, they identified that hypertension, rather than PFO, showed a close relationship with recurrence [203]. Therefore, multidisciplinary consultation and discussion are critical for accurate diagnosis, appropriate patient selection, and shared decision‐making regarding PFO closure in older patients. In particular, prolonged and intensive cardiac monitoring is essential to rule out subclinical AF in older patients with CS. Currently, several RCTs are ongoing to compare medical treatment versus PFO closure in this age group (Table 1) [188]. We look forward to more definitive evidence regarding the role of PFO closure in older patients. As life expectancy continues to increase globally, defining the optimal management strategy for this growing population will become increasingly important.

#### 7.3.2 Pediatric Population

Similar to adults, PFO is associated with certain clinical syndromes in the pediatric population, particularly CS and migraine, which affect their health. Although pediatric stroke is relatively rare, it is associated with poor neurological prognosis. Children with sickle cell disease, an inherited hemoglobinopathy affecting more than 300,000 newborns annually [204], are predisposed to a higher risk of paradoxical embolization when a PFO coexists [205]. Among children with CS, the prevalence of PFO is also higher compared with healthy children, a pattern similar to that observed in adults [206]. Unlike in adults, TEE examination is considerably limited in pediatric applications due to the requirement for anesthesia. In contrast, TTE, especially cTTE, has been considered the optimal examination for diagnosing PFO in this population due to the excellent transthoracic windows and the noninvasive characteristics [205, 206].

In children with PFO‐related CS, closure may be considered to reduce the risk of recurrence, although current evidence is largely limited to case reports [206]. Zhang et al. reported the case of a 7‐year‐old boy who experienced multiple cerebral infarctions attributed to PFO. After PFO closure, the boy did not suffer any new cerebral infarction during 1 year of follow‐up [207]. More evidence has focused on migraine outcomes. Several retrospective studies suggest that PFO closure may improve migraine symptoms in pediatric patients, with reductions in migraine scores [208, 209]. In addition, improvements were also observed in children with unexplained dizziness [208]. Importantly, the above studies all demonstrated a high success rate, few or no procedure‐related complications, as well as residual shunt, indicating the safety of this procedure in children as in adults [208, 209]. Therefore, these findings documented that PFO closure may be considered for children with neurological diseases, especially stroke.

Unfortunately, all of the available studies are retrospective. High‐quality and robust evidence from large‐scale RCTs regarding the management of PFO in the pediatric population is lacking, and no unified clinical guidelines or consensus recommendations have been established. For asymptomatic children with incidentally discovered PFO, further treatment or follow‐up is not generally recommended [205]. However, in high‐risk pediatric populations, including children with sickle cell disease whose risk of stroke is further increased when accompanied by PFO, screening for PFO and appropriate evaluation may be especially crucial [206]. In special cases involving CS or TIA, directly applying adult guidelines to the pediatric population is inappropriate given their unique characteristics. Clinically, a principle of multidisciplinary collaboration and individualized treatment should be adhered to ensure the most appropriate management plan. In the future, multicenter RCTs are needed to establish optimal management strategies for PFO in the pediatric population, particularly regarding PFO closure.

#### 7.3.3 Pregnancy

Although ischemic stroke during pregnancy is rare, both PFO and pregnancy are recognized as independent risk factors for stroke in young adults. Marked physiological changes in preload, cardiac output, and systemic vascular resistance may influence the extent of RLS in pregnant individuals with a PFO. The hemodynamic and hormonal changes associated with pregnancy may further contribute to PFO‐related stroke. Furthermore, pregnancy is characterized by a hypercoagulable state, which increases the risk of venous thromboembolism compared with the nonpregnant state. The presence of a PFO may confer a theoretical risk for paradoxical embolism, thereby increasing the risk of PFO‐related stroke [210]. Evidence suggests that the risk of PFO‐related stroke rises during pregnancy, particularly in the presence of venous thromboembolism, and may persist into the postpartum period [142]. A case report described a young woman in her third trimester who developed an acute ischemic stroke attributed to a PFO. Subsequent PFO closure was performed in the postpartum period, underscoring the importance of considering PFO as a potential cause of stroke during pregnancy [211]. In a meta‐analysis evaluating adverse pregnancy outcomes in 28 pregnant women with PFO‐associated complications, paradoxical embolic events occurred in 60% of cases during pregnancy, 11% during delivery, and 29% postpartum. Ischemic events accounted for the majority (89%), with ischemic stroke being the most common (57%), followed by TIA (14%), AMI (7%), and branch RAO (11%). PFO closure was performed in 42% of patients during pregnancy, 14% postpartum, while 25% received medical therapy alone [212]. Despite these observations, data regarding PFO in pregnant women remain limited, and significant knowledge gaps persist. Currently, no guidelines address stroke prevention strategies in this population. The safety and efficacy of PFO closure during pregnancy remain uncertain, and existing guidelines do not recommend closure during pregnancy, although this procedure has been reported in the pregnant population. At present, for PFO‐related stroke occurring during pregnancy, expert consensus continues to recommend initial anticoagulation therapy, with deferring PFO closure until after delivery [210]. Overall, further studies are needed to establish evidence‐based management strategies for pregnant women with PFO, with careful consideration of both maternal and fetal safety.

#### 7.3.4 Athletes

Whether PFO closure can be similarly applied to special populations, such as athletes, and whether sports participation influences long‐term prognosis after closure, remains to be fully explored. Recently, D'Ascenzi et al. analyzed 36 athletes and 40 nonathletes who underwent PFO/ASD closure and found no significant differences between the two groups in either procedural‐related complications or bleeding events during a long‐term follow‐up of over 10 years [213]. These findings suggest that PFO closure appears to be safe in athletes, and sports participation did not increase the bleeding risk following the procedure. Further large‐scale studies are still warranted to validate these findings.

### 7.4 Issues to be Addressed After PFO Closure

#### 7.4.1 Arrhythmias

PFO closure has a high procedural success rate and a favorable safety profile, with relatively low rates of procedural complications and adverse events [214]. Potential procedural complications include arrhythmias, cardiac erosion and tamponade, device embolization, infective endocarditis, peripheral vascular complications, and residual shunt [215, 216, 217]. Among these, atrial arrhythmia is the most common complication after PFO closure, which may be associated with the direct impacts of the implanted device on the atria. First, the presence of an intracardiac device may affect atrial myocardium, predisposing to electrical instability. Evidence suggests that a larger left atrial disk of the device is related to a higher prevalence of atrial arrhythmia [218]. Second, the device‐induced inflammatory responses within the atrial septum may also be contributory [219, 220]. Third, atrial mechanical stretch triggered by device deployment may be another possible mechanism [221]. In a prospective study, implantable loop recorder monitoring was conducted in patients at higher risk for AF after PFO closure. Supraventricular arrhythmia was diagnosed in approximately 28.9% of patients, with older age, device left disc diameter, and male sex as independent risk factors [222]. Similarly, in the AFLOAT study enrolling 186 patients who received PFO closure, 53 (28.5%) patients developed atrial arrhythmia during a 6‐month follow‐up, and 86.8% of these events occurred within the first month [218]. Given that the study population in the AFLOAT study was older, predominantly male, and had a higher prevalence of hypertension and obesity, these factors may contribute to the development of atrial arrhythmias [220]. Although most arrhythmias occur within the first month, late‐onset ones also exist. In a study by Giovachini et al., 21.4% (50 out of 234) of patients developed supraventricular arrhythmias more than 28 days after PFO closure. Early supraventricular arrhythmia burden, male sex, and larger left atrial disk diameter were identified as independent predictors of late arrhythmia [216].

AF accounted for the most common type of arrhythmia and adverse event observed after PFO closure [217]. In the AFLOAT study, AF accounted for 89.1% of all atrial arrhythmias, with the remaining 4.9% and 6.0% being atrial flutter and atrial tachycardia, respectively [218]. The overall incidence of postprocedural AF is generally low, ranging from 1.4 to 6.6% across most studies [10, 153, 154, 166, 179, 201, 214]. The differences in study design, monitoring strategies, device types, and duration of follow‐up may lead to heterogeneity in reported data. However, in some reports, the incidence exceeds 9% and may reach up to 12.54% [151, 223, 224]. It is also possible that some patients have undiagnosed pre‐existing AF before PFO closure, which leads to an overestimation of the true incidence of postprocedural AF [221]. The precise incidence of AF following PFO closure remains to be determined and requires confirmation from large‐scale epidemiological studies.

From a temporal perspective, most AF is typically transient and occurs early after the procedure, primarily within the first month or within 45 days [214, 221, 225]. The incidence subsequently declines, which may be related to progressive device endothelialization. Currently, there is no evidence reporting that PFO closure increases the long‐term risk of AF [221]. Although the majority of observational studies and meta‐analyses have indicated that patients who underwent PFO closure have an increased incidence of AF compared with those receiving medical therapy alone [11, 146, 149, 150, 225], inconsistent opinions also exist, reporting no significant difference in AF occurrence after the 1‐month follow‐up, a time that is beyond the early postprocedural period [225].

Generally, postclosure AF is often transient and considered benign and has minimal long‐term impact on cardiac function [221, 226]. However, new‐onset AF remains a clinical concern, as it may adversely affect short‐term outcomes and increase the risk of thromboembolic events, including ischemic stroke, due to embolism from the LA [220]. The pathophysiological mechanisms of AF following PFO closure remain incompletely understood. It is speculated that some procedural factors may be involved, including device‐related interference, local irritation and inflammation of the atrial septum, tissue stretch, and nickel hypersensitivity [221].

Several studies have shown that PFO closure may be associated with a higher incidence of AF in patients over 60 years old [141, 201]. However, beyond age, other factors, such as alcohol consumption and a larger left atrial volume, may also be associated with an increased risk of developing AF after PFO closure, confounding the relationship between age and postprocedural AF [223]. A meta‐analysis performed by Spyropoulou et al. found no age‐dependent AF occurrence following PFO closure [225]. Similarly, another meta‐analysis including 7275 elderly patients (≥55 years) with CS also found no significant difference in new‐onset AF between patients undergoing PFO closure and those receiving medical therapy alone [227]. Similar findings were reported by another meta‐analysis involving patients aged over 60 years, which reported that the occurrence of new‐onset AF was comparable between patients undergoing PFO closure and those receiving medical therapy alone. However, within the PFO closure subgroup, the older patients (≥60 years) exhibited a higher risk of new‐onset AF compared with younger individuals [199]. These findings suggest that postoperative monitoring for AF in older patients may be necessary for early detection and timely intervention. After all, although postclosure AF is well tolerated when promptly treated, delayed recognition may increase the risk of stroke and hospitalizations [220].

Several studies have attempted to identify predictors of AF after PFO closure. It has been reported that left atrial strain may be a valuable imaging biomarker for predicting AF occurrence [221]. In addition, Crea et al. found a significant difference in the MVP (Morphology‐Voltage‐P wave duration) ECG risk score between patients with or without AF after PFO closure, suggesting that it may be an independent predictor of early postprocedural AF [228]. Although it is well recognized that preventing the onset of arrhythmias, particularly AF after PFO closure, may improve patient outcomes, effective preventive methods remain limited. In the AFLOAT study, the use of flecainide did not reduce the incidence or duration of atrial arrhythmia after PFO closure [218]. Therefore, large‐scale, prospective, multicenter studies are needed to define risk stratification strategies and to investigate the long‐term clinical impact of postprocedural AF, particularly stroke risk.

#### 7.4.2 Residual Shunt

Residual shunt, occurring at an incidence of 20–25% across different studies, is an important concern following percutaneous PFO closure [229, 230, 231]. It is directly associated with an increased risk of recurrent ischemic stroke or TIA [232, 233]. Moreover, a dose–response relationship has been observed, with larger shunts conferring a higher risk [233]. Therefore, reducing or eliminating the residual shunt is considered to effectively decrease stroke risk.

The degree of residual shunt may be associated with PFO anatomical features. Several studies have explored potential predictors. In a study by Mi et al., 24% (22 out of 92) of patients presented significant residual shunt at 12‐month follow‐up after the PFO procedure, more likely in individuals presenting with a thicker secondary septum thickness (>6.55 mm) or longer tunnel length (>10.10 mm) [229]. Similarly, Gao et al. found that atrial cardiopathy, body mass index, and ASA were independent predictors of residual shunt [234], while another study enrolling 527 patients reported a higher incidence of residual shunt in individuals with ASA and larger PFO size [235]. However, these findings are not consistent across studies. In a single‐center study of 103 patients successfully undergoing PFO closure, 21% of the patients presented significant residual shunt. Additional anatomical characteristics, including aortic rim, tunnel length, and IVC–PFO tunnel angle, were also associated with residual shunt [230]. In contrast, Nakayama et al. found no significant association between residual shunt and PFO size, tunnel length, or device size, although the association with ASA still existed [231]. Differences in study design, inclusion criteria, and imaging assessment standards may lead to discrepancies in the results. Overall, the mechanisms underlying residual shunting remain incompletely understood and may involve device‐related leakage, pulmonary arteriovenous malformations, uncovered septal fenestrations, or small ASD [236].

Does the presence of a large residual shunt warrant immediate intervention? Evidence indicates that residual shunts often decrease or resolve spontaneously over time [233]. In a retrospective study of 498 patients who underwent PFO closure, residual shunt was observed in 35% of patients at 6 months postprocedure, dropping to just 19% at 12 months [233]. Similarly, an observational study from Slovenia involving 355 patients reported an increase in complete closure rates, defined as no bubbles on cTEE, from 64% immediately after intervention to 80% at 6 months [236]. In another cohort of 227 patients, the overall incidence of residual shunt decreased from 27.3% at 6 months to 12.3% at 12 months, with small, moderate, and large residual shunts all dropping by half. These indicate that more than half of residual shunts resolved between 6 and 12 months following closure [237].

Accordingly, immediate reintervention for residual shunt is not routinely warranted in most cases. However, in patients with persistent large residual shunts beyond 1 year postintervention, reintervention may be considered due to a higher risk of recurrent CVEs [233]. Secondary transcatheter closure using an additional device is an appropriate strategy due to its efficiency and minimal invasiveness. In a study by He et al., 12 out of 14 patients (85.7%) with large residual shunts achieved complete success following residual shunt closure using PFO occluders, ASD occluders, or an ADO‐II device [238]. Luo et al. reported successful treatment of residual shunt with a biodegradable PFO occluder in a 52‐year‐old woman with recurrent stroke and migraine after PFO closure [239]. Despite these advances, evidence addressing the management of residual shunt remains limited, and further large‐scale studies are needed to define indications for reintervention.

## 8 Conclusions and Perspectives

PFO is a highly prevalent and heterogeneous cardiac abnormality with increasing recognized clinical significance. Paradoxical embolism and in situ thrombus are the principal pathological mechanisms underlying PFO‐related cerebral and systemic embolic events. Substantial evidence has established the causal role of PFO in CS and transcatheter closure for secondary prevention in patients with CS. In contrast, the associations between PFO and other neurological or hypoxic disorders remain unclear, and even controversial. Given that these diseases are often attributed to multiple factors beyond PFO, distinguishing the pathogenic from the incidental PFO remains a main challenge in contemporary PFO management.

Multiple imaging evaluations, careful exclusion of other possible etiologies, and appropriate risk stratification may contribute to identifying the patients who are most likely to benefit from the closure, avoiding unnecessary intervention. Nevertheless, the feasibility and accuracy of current diagnostic and predictive tools in special populations, including elderly individuals, pediatric patients, pregnant women, and athletes, need further investigation. In recent years, advances in device technology have facilitated shifting the therapeutic paradigm into a more physiologically compatible state, aiming to minimize the cardiac retention of occlusion materials and reduce device‐related postoperative complications, although long‐term efficacy and safety remain uncertain. Moreover, despite the favorable procedural success and safety profile of current closure surgery, there is still a need for long‐term surveillance against complications such as arrhythmias and residual shunts. Meanwhile, the optimal antithrombotic and long‐term follow‐up strategy after the surgery has not yet been standardized.

At present, apart from the three RCTs in stroke secondary prevention, no other RCTs have yielded encouraging results for PFO closure. Therefore, future focus should be put on generating high‐quality evidence across different diseases, with newly developed devices, and among special populations. Ultimately, the future of PFO management lies not in closing more PFOs, but in identifying the PFOs that truly matter and require intervention, and advancing toward a precision medicine‐based therapeutic era.

## Author Contributions

X.M., C.Z., and Y.Z. contributed to the conception and design of the work. X.Z., G.S., and H.D. collected the literature and information. L.M. and X.M. wrote the manuscript. All authors have read and agreed to the published version of the manuscript.

## Funding

This work was supported by the grants of the National Natural Science Foundation of China (81970319—X.M., 82500465—L.M., 82430015—Y.Z., 82527803—Y.Z.), State Key R&D Program of China (2021YFF0501403—C.Z.), the Taishan Scholars Program of Shandong Province (ts201511091—C.Z., tsqn202103170—X.M.), National Natural Science Foundation of Shandong (ZR2024QH520—L.M., ZR2024ZD09—C.Z.), and Shandong Postdoctoral Science Foundation (SDBX202302026—L.M.).

## Ethics Statement

The authors have nothing to report.

## Conflicts of Interest

The authors declare no conflicts of interest.

## Data Availability

The authors have nothing to report.
